# Ultra-wideband integrated photonic devices on silicon platform: from visible to mid-IR

**DOI:** 10.1515/nanoph-2022-0575

**Published:** 2023-01-18

**Authors:** Xuhan Guo, Xingchen Ji, Baicheng Yao, Teng Tan, Allen Chu, Ohad Westreich, Avik Dutt, Cheewei Wong, Yikai Su

**Affiliations:** State Key Laboratory of Advanced Optical Communication Systems and Networks, Shanghai Jiao Tong University, Shanghai, China; John Hopcroft Center for Computer Science, School of Electronic Information and Electrical Engineering, Shanghai Jiao Tong University, Shanghai 200240, China; Key Laboratory of Optical Fibre Sensing and Communications (Education Ministry of China), University of Electronic Science and Technology of China, Chengdu, China; Fang Lu Mesoscopic Optics and Quantum Electronics Laboratory, University of California, Los Angeles, CA, USA; Applied Physics Division, Soreq NRC, Yavne 81800, Israel; Mechanical Engineering, and Institute for Physical Science and Technology, University of Maryland, College Park, USA

**Keywords:** heterogeneous integration, mid infrared, silicon photonics, visible, wide bandgap

## Abstract

Silicon photonics has gained great success mainly due to the promise of realizing compact devices in high volume through the low-cost foundry model. It is burgeoning from laboratory research into commercial production endeavors such as datacom and telecom. However, it is unsuitable for some emerging applications which require coverage across the visible or mid infrared (mid-IR) wavelength bands. It is desirable to introduce other wideband materials through heterogeneous integration, while keeping the integration compatible with wafer-scale fabrication processes on silicon substrates. We discuss the properties of silicon-family materials including silicon, silicon nitride, and silica, and other non-group IV materials such as metal oxide, tantalum pentoxide, lithium niobate, aluminum nitride, gallium nitride, barium titanate, piezoelectric lead zirconate titanate, and 2D materials. Typical examples of devices using these materials on silicon platform are provided. We then introduce a general fabrication method and low-loss process treatment for photonic devices on the silicon platform. From an applications viewpoint, we focus on three new areas requiring integration: sensing, optical comb generation, and quantum information processing. Finally, we conclude with perspectives on how new materials and integration methods can address previously unattainable wavelength bands while maintaining the advantages of silicon, thus showing great potential for future widespread applications.

## 1 Introduction

Silicon photonics has gained great success in many areas owing to the high-density feature and compatibility with complementary metal-oxide-semiconductor (CMOS) fabrication technique where low cost is necessary, starting from telecom application as a representative example in the 1.5-μm wavelength band. However, the transparency window of silicon is from 1.1 μm to 8.5 μm due to its material bandgap, and hence, silicon is unsuitable for some emerging applications which require visible or mid infrared (mid-IR) wavelengths, such as quantum optics [1, 2], precision metrology and spectroscopy [3, 4], red–green–blue (RGB) displays [5], atomic clocks [6], computing [7, 8], remote sensing [9], etc.

It is very challenging for a single-material system to offer solutions to diverse application requirements spanning from the visible to the mid-IR. Wide bandgap photonic circuits may rely on heterogeneous integration of multiple material systems, while the integration is desired to be compatible with wafer-scale fabrication processes starting from silicon substrates. Incorporation of new materials with silicon is possible either with alternative moderate-index-contrast systems that are manufacturable in the same CMOS environment (Silica, Si_3_N_4_, etc.) or through heterogeneous integration with other wide bandgap materials (LN, Ta_2_O_2_, Al_2_O_3_, AIN, 2D materials, etc.). These methods can reach the previously unattainable wavelength bands by using wide band gap semiconductors, while maintaining the advantages of silicon, thus showing great potential for future research and applications. Some other wide bandgap materials are also drawing great attentions. For example, the chalcogenide glass that replaces oxygen with heavier chalcogens is one of wide bandgap semiconductor optical materials [10], which exhibits broadband transparency, high and continuously tunable refractive indices (*n* ∼2–3.5) and large Kerr nonlinearity (∼10^−18^ m^2^ W^−1^). Hybrid silicon-chalcogenide glass photonic integrated circuits [11] as well as photonic integration of 2D materials using chalcogenide glass [12] have been demonstrated. Germanium (Ge) is also a promising mid-IR optical material with high refractive index (*n* = 4.0) and a large transparency range up to 14.6 μm [13]. Due to the page limit, chalcogenide glass and Ge materials on silicon are not covered in this review.

In this paper, we review the latest development of ultra-wideband integrated photonic devices based on the silicon platform from visible to mid-IR, including material properties, fabrication, structures and applications, so as to illustrate the development towards new applications. The review paper is organized as follows: Firstly, we will start with wideband materials and integrated photonic devices on the silicon platform, including their optical properties, representative structures, general fabrication process and the latest research progress of the functional wideband components. Secondly, we will focus on the emerging applications from the perspective of different operating wavelength bands. Finally, we will discuss the challenges toward realization of ultra-wideband integrated systems, motivating the need for wide bandgap integrated photonic devices. We hope to address the latest advances in these wideband silicon-based devices and provide some vision for the future of next-generation integrated photonics.

## 2 Materials and integrated photonic devices on silicon platform

Benefiting from the scaling of mature CMOS-compatible foundries and the associated toolkits first developed for the microelectronics ecosystem, silicon photonics has been thriving from laboratory research and development (R & D) into commercial production endeavors such as datacom and telecom, where high-volume and low-cost foundry models are critical. Silicon-on-isolator (SOI) is undoubtedly the leading material platform, which has successfully realized various high-performance components and characteristic applications, mainly in a spectral range of 1.1–3.8 μm, where both silicon and silica are transparent [14], especially near the communication windows of 1.31 (O-band) and 1.55 μm (C-band) [15].

**Figure 1:**
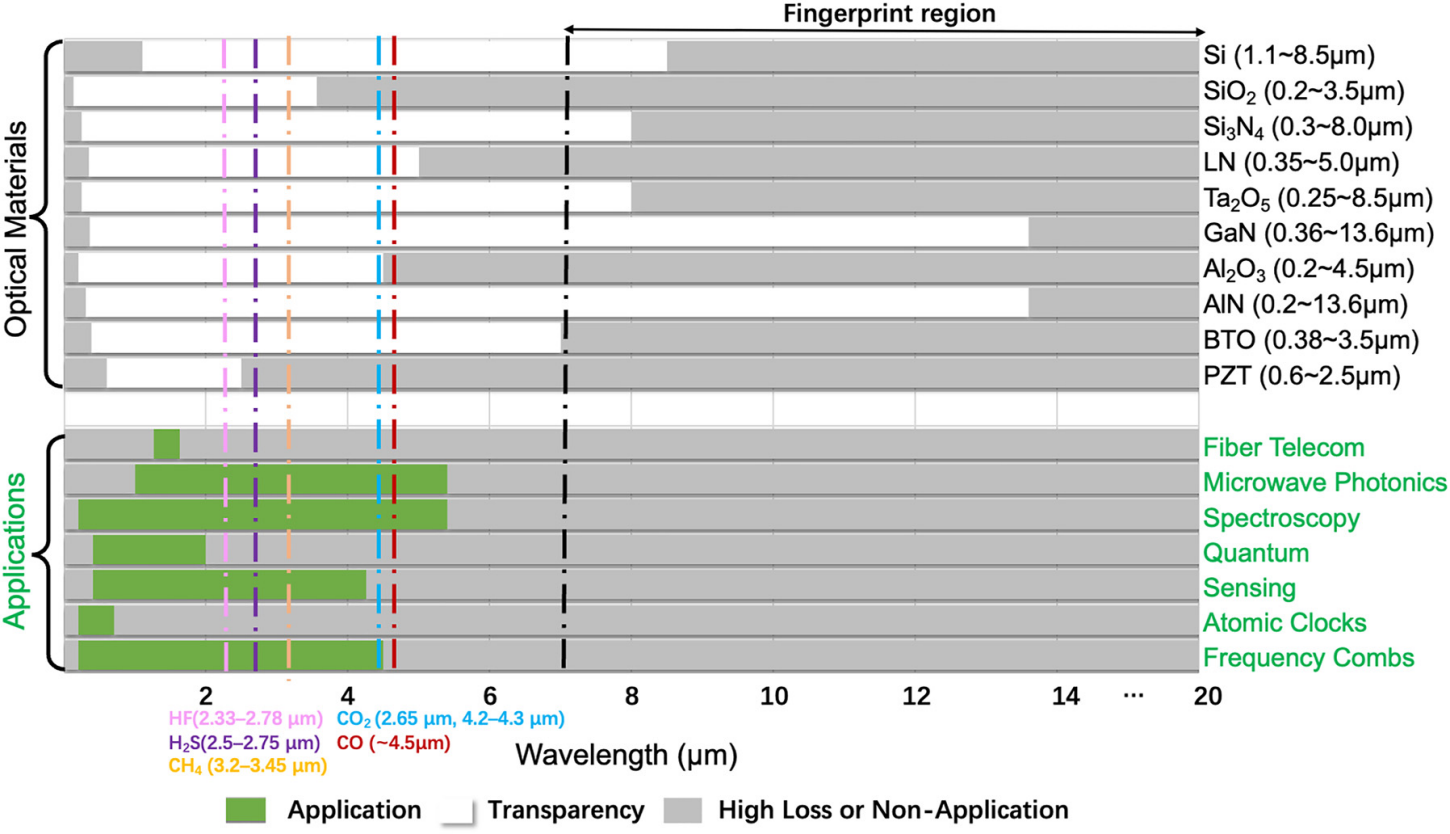
Transparency windows of different photonic materials and applications over various spectral windows.

The range of transparency determines the potential application fields of a material platform. Recently, a wide range of emerging applications have driven the need for new wide-bandwidth photonic integration other than telecom and datacom, as illustrated in Figure 1, which require low optical losses extending from the visible (∼400 nm–700 nm) to the mid-infrared (mid-IR, ∼20 μm). For instance, mid-IR is a very important wave band for photonics which encompasses multiple atmospheric sensing windows covering the fingerprint region (7–20 μm) as well as the primary absorption peaks/bands of typical chemical and biological molecules [16], including CO_2_ (2.65 μm, 4.2–4.3 μm), CH_4_ (3.2–3.45 μm) and CO (∼4.5 μm), etc., and some other toxic gases such as HF (2.33–2.78 μm) and H_2_S (2.5–2.75 μm) etc. These windows are of huge interest to spectroscopic sensing, thermal imaging and infrared homing, etc.

These new applications require different on-chip properties within the wavelength range of interest, either low linear loss for the passive components (i.e. waveguides, splitters, filters, and demultiplexers), or the nonlinearity and dispersion properties (i.e. electro-optic, optoelectronic, and thermo–optic) for active components (i.e. modulators, phase shifters, photodiodes, and comb generators), or piezoelectric property (i.e. microelectromechanical systems (MEMS)). However, application spaces for silicon photonics in these wavelength ranges are still immature, mainly limited by the nontransparent wavelength window, or high waveguide loss, or the inefficient nonlinearity for high power handling. Therefore, in order to expand the operating wavelength ranges and applications, there is an increasing trend in scaling up silicon-based devices [17]. To realize the ultra-wideband applications and leverage the successful silicon photonic integrated circuits technologies, the wideband integrated photonic devices on silicon platform will most likely support heterogeneous integration of a range of passive and active functionalities, as well as the ability to enable low loss components and high integration density, the realization of high-performance passive, active and nonlinear components, and some unique material properties to realize novel devices. Various wide bandgap semiconductor optical materials with low losses have beenr demonstrated that can support wafer-scale o heterogeneous integration, of which the most reported materials include silicon family materials such as silica (SiO_2_) and silicon nitride (Si_3_N_4_), or the promising wideband optical material that can be integrated on silicon platform through various integration approaches, i.e. 2D materials, aluminum nitride (AlN), gallium nitride (GaN), aluminum oxide (Al_2_O_3_), tantalum pentoxide (Ta_2_O_3_), lithium niobate (LN), barium titanate (BaTiO_3_–BTO), and lead zirconate titanate (PZT).

The typical transparency windows of different photonic materials and applications over various spectral windows are shown in Figure 1.

### 2.1 Silicon-family materials

Silicon-family materials based on mature silicon photonic platforms mainly include silicon, silicon nitride, and silica. As silicon’s crystal structure has inversion symmetry (hence classified as “centro-symmetric” without the second order nonlinear response (Pockels effect)), the silicon-family materials do not show the linear electro-optic (EO) effect when an electric field is applied. Most silicon-family material and related application fields focus on their superior passive performances [15]. The optical properties of the silicon family are summarized in Table 1.

**Table 1:** Optical properties of the silicon family [14, 15, 18].

Parameter	Si	Si_3_N_4_	SiO_2_
Bandgap	1.14 eV (1.09 μm)	5.0 eV (0.25 μm)	9.3 eV (0.13 μm)
Refractive index at 1.55 μm	3.487	∼2.0	1.444
TO coefficient (K^−1^)	1.86 × 10^−4^	2.45 × 10^−5^	0.95 × 10^−5^
TPA coefficient	9 × 10^−12^	0	–
Kerr coefficient (m^2^W^−1^)	4.4 × 10^−18^	2.4 × 10^−19^ [19]	2.2 × 10^−20^ [20]
Typical waveguide loss around 1.55 μm (dB/cm)	1 ∼ 1.5	0.001 ∼ 0.5	<0.1
Cladding	Silica/air	Silica	Air
Pockels effect	–	–	–

#### 2.1.1 Silicon

**Figure 2:**
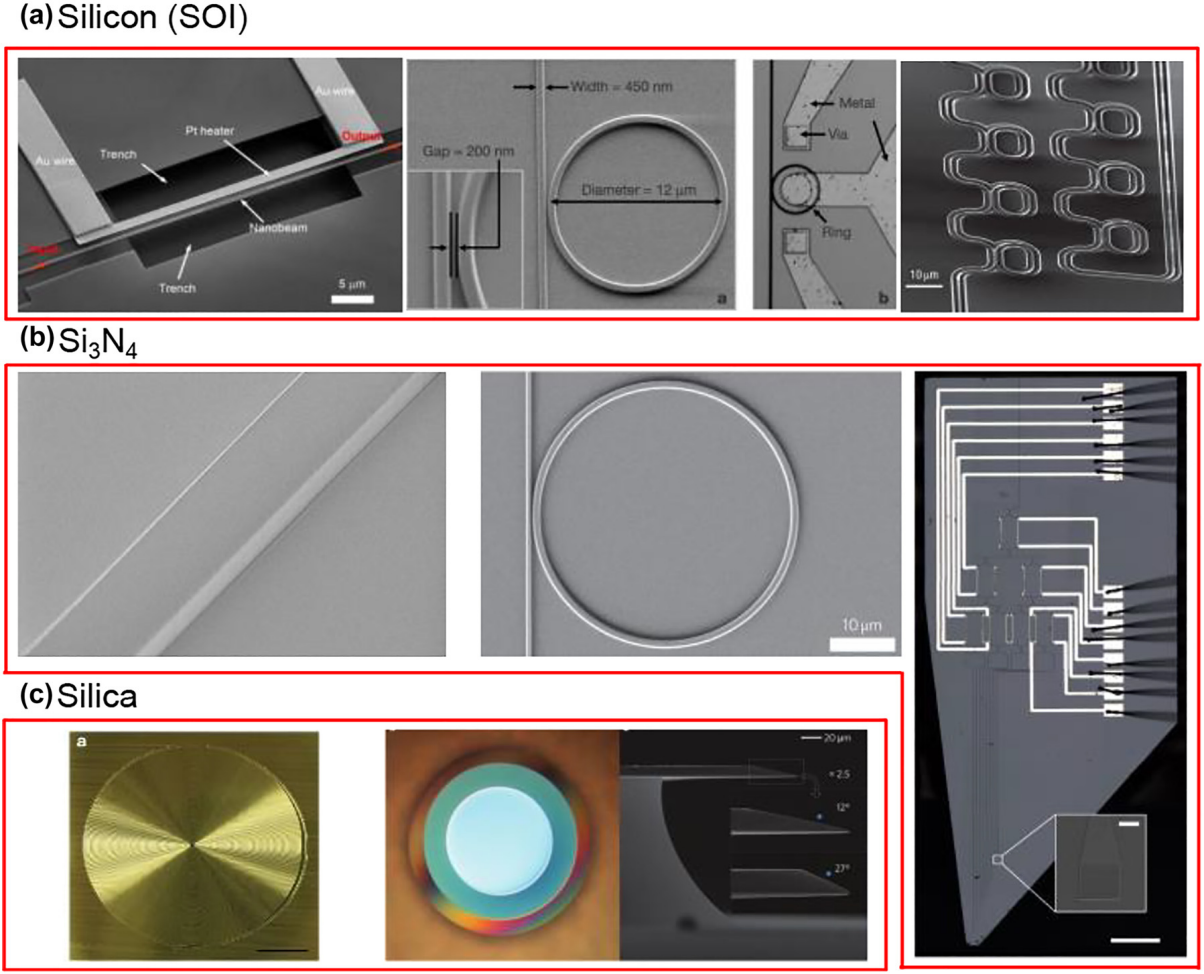
Typical devices of silicon-family materials. (a) Silicon: a single-resonance silicon nanobeam filter [22]; a micrometer-scale silicon electro-optic modulator [23]; an on-chip silicon waveguides and cascaded microring resonators based optical delay line [24]. (b) Silicon nitride: scanning electron microscopy (SEM) image of a fabricated silicon nitride waveguide [50]; a SEM of a silicon nitride optical parametric oscillator [51]; a silicon nitride switch [52]. (c) Silica: a silica spiral delay line [87]; micrographs and SEM of a chemically etched ultrahigh-Q wedge-resonator on a silicon chip [88]. [(a) Reproduced with permission from reference [22]. Copyright © 2018, optical society of America; reproduced with permission from reference [23]. Copyright © 2005, nature publishing group; reproduced with permission from reference [24]. Copyright © 2005, nature publishing group; (b) reproduced with permission from reference [50]. Copyright © 2017, optical society of America; reproduced with permission from reference [51]. Copyright © 2009, nature publishing group; reproduced with permission from reference [52]. Copyright © 2020, nature publishing group; (c) reproduced with permission from reference [87]. Copyright © 2012, nature publishing group; reproduced with permission from reference [88]. Copyright © 2012, nature publishing group].

Silicon exhibits a very high refractive index of ∼3.4, making it possible to realize compact footprint and densely-integrated devices on a commercial SOI wafer. Silicon material itself shows low absorption in the wavelength range of 1.1–8.5 µm, which covers the near-infrared (near-IR) and mid-infrared (mid-IR) regions. However, the prevailing SOI platform for silicon photonics is mainly transparent between 1.1 μm and 4 μm [14]. Below 1.1 µm, silicon strongly absorbs the guiding light, and its lower limit is determined by the narrow bandgap, but it can be used for photodetection. Beyond ∼4 µm, for silicon devices with buried oxide (BOX) or cladding layer, there is a sharp onset of mid-IR absorption of the in the range of 2.6∼2.9 μm and beyond 3.6 μm [21]. Some typical devices of silicon are shown in Figure 2(a) with two examples: a silicon nanobeam filter with an ultrahigh thermo-optic tuning efficiency of 21 nm/mW over a wide continuous tuning range of ∼43.9 nm [22], a micrometer-scale silicon electro-optic modulator with a modulation speed of 1.5 Gbit/s [23] and an on-chip optical delay line based on silicon waveguides and cascaded microring resonators with group delays exceeding 500 ps and a footprint less than 0.09 mm^2^ [24].

Due to the fabrication compatibility with CMOS pilot lines and good optical properties, silicon photonics based on SOI platform has predominantly been used to demonstrate components for short and long haul communication systems in near-IR range (1.26∼1.63 μm). Fully etched silicon strip waveguides have a typical waveguide loss of ∼1.5 dB/cm for TE polarized light in the C-band, and the loss can be further reduced to ∼0.7 dB/cm for partially etched rib waveguides [25]. Amongst other functional components [25, 26] include various high-speed transceivers [27], modulators [28], wavelength filters for wavelength division multiplexing (WDM) systems [29] and photodetectors [30]. Starting from the great success in telecommunications, other applications such as optical spectrometers [31], optical neural networks [32, 33], and nonlinear applications [34] have also been demonstrated. Vast reviews of silicon photonic devices and applications in the near-IR band can be found in [15, 25, 35]. Silicon has a large third-order non-linearity with a large Kerr index (4.4 × 10^−18^ m^2^ W^−1^), however, the presence of two-photon absorption (TPA) resulting from its small bandgap of 1.1 eV and induced free-carrier absorption make it inefficient for nonlinear applications or other high power handling applications [25] in the telecommunication band. Silicon is also very sensitive to temperature variations due to the high thermo–optic (TO) coefficient of 1.86 × 10^−4^ K^−1^. This TO effect has been utilized for implementing tunable filters, low-speed thermo-optic switches, couplers with arbitrary coupling ratios or to compensate fabrication-induced phase errors on delay lines or wavelength sensitive filters.

For wavelengths beyond ∼2.2 μm (i.e. twice the bandgap wavelength of 1.1 μm), there is a set of gases characteristic absorption peaks in this band [16]. TPA problem in silicon is also drastically reduced, making silicon waveguide structures ideal for implementing new scenarios, such as spectroscopic, sensing applications, and nonlinear optical devices in the mid-IR [36–38]. In order to fully utilize the wavelength transparency window of silicon on an SOI platform in the mid-IR range, free-standing silicon device structures fabricated by under-etching the BOX are reported [39, 40]. Various functional mid-IR devices based on silicon have been demonstrated, including waveguides, resonators, grating couplers, multi-mode interferometers (MMIs) and Mach–Zehnder interferometers (MZIs), arrayed waveguide gratings (AWGs), modulators, frequency combs and light sources, etc. For passive components, suspended waveguides with low-dispersion (±100 ps/nm/km) over a wavelength range of 2∼8 μm has been first demonstrated in 2012 [39] with a minimum waveguide loss of 3.0 ± 0.7 dB/cm, and a suspended ring resonator with Q factors of ∼10,000 and ∼8100 for near-IR and mid-IR were also reported. A subwavelength grating (SWG) coupler with a 24.7% coupling efficiency at 2.75 μm [40] and a 1 × 2 MMI-based wavelength demultiplexer with a low insertion loss of 1.2 dB at 2 μm [41] were demonstrated. For active components, a mid-IR TO modulator has been reported [42] using a spiral-based asymmetric MZI with aluminum heaters. The modulator operates at 3.8 μm with a 30.5-dB-high modulation depth, a 3-dB bandwidth of 23.8 kHz and a switching power of ∼47 mW. The first free-carrier injection-based mid-IR electro-optic (EO) modulator in SOI at 2.165 μm with a modulation speed up to 3 Gbit/s was demonstrated in 2012 [43]. The modulator has a *V*_*π*_·*L* of 0.12 V mm with an extinction ratio of 23 dB. For nonlinear devices, a SOI ring-resonator–based on-chip frequency comb has been shown, with a spectrum spanning from 2.1 to 3.5 μm and a frequency spacing of about 127 GHz [44]. The ring resonator was fabricated using an etchless process, showing a loaded *Q*∼220,000 and an intrinsic *Q*∼590,000, respectively. In 2015, an octave-spanning mid-IR (1.5–3.3 μm) frequency comb was generated in a silicon nanophotonic wire waveguide on a room-temperature-operating CMOS-compatible chip [45]. In 2022, a single-mode mid-IR laser at 3.4 μm with a wide tuning range of 54 nm using a tunable high-Q silicon microring cavity and a multi-mode interband cascade laser(ICL) [46] was reported. The single-frequency lasing power reaches 0.4 mW via self-injection locking, with upper-bound effective linewidth estimated to be 9.1 MHz and a side mode suppression ratio of 25 dB. This chip-scale and tunable single mode high power mid-IR light source may be expanded to longer wavelength quantum-cascade lasers and lead to the development of compact, high-performance mid-IR sensors for spectroscopic applications. Because of the reduced nonlinear absorption, other nonlinear photonic functions such as parametric amplification [47] and wavelength conversion [48] have also been demonstrated in the short-wave IR and mid-IR. By engineering the waveguide cross-section and optical mode interaction with the absorptive cladding oxide to reduce loss at mid-IR wavelengths, a microring resonator with an ultrahigh Q factor of 10^6^ at wavelengths from 3.5 to 3.8 μm has been reported, which was used for optical parametric oscillation with a low threshold of 5.2 mW [37]. These works pave the way for a wide working band from near-IR to mid-IR for silicon photonic devices.

#### 2.1.2 Silicon nitride

Silicon nitride (Si_3_N_4_) is emerging as a promising material to complement silicon at wavelengths below 1.1 µm due to its wide optical bandgap and ultralow absorption loss. By changing the N/Si ratio, silicon nitride has an optical bandgap that can vary from 2.7 to 5.0 eV, covering a wide transparency range from visible to mid-IR (∼300 nm–8 μm). It also has a moderately high nonlinear refractive index (*n*_2_ = 2.4 × 10^−19^ m^2^/W) which is ten times higher than silica, and most importantly semiconductor mass manufacturing compatibility [15, 19, 49]. By leveraging mature silicon fabrication techniques, it shows great promise for delivering low-cost, high-yield, small form-factor, and low-power consumption integrated photonic components. Some typical devices of Si_3_N_4_ are shown in Figure 2(b). Some typical devices of Si_3_N_4_ are shown in Figure 2(b) including a fabricated silicon nitride waveguide with smooth surfaces [50], a silicon nitride microring resonator coupled to a bus waveguide as an optical parametric oscillator [51] and a nanophotonic switch operating in the visible spectral range for sub-millisecond deep-brain optical stimulation [52]. There are two most common methods to deposit Si_3_N_4_ films, namely low-pressure chemical vapor deposition (LPCVD) and plasma-enhanced chemical vapor deposition (PECVD). These chemical vapor deposition processes depend on both chemical reactions and gas flow dynamics. In the LPCVD process, the important variables are pressure, temperature, flow rate, and gas ratio. In the PECVD process, in addition to these variables, RF power can also be varied. LPCVD Si_3_N_4_ films are deposited at high temperatures around 800 °C, and PECVD films are deposited at much lower temperatures around 300 °C–400 °C. While lower temperature has the benefit of enabling safe deposition on temperature-sensitive materials and devices, it has the disadvantages of producing films with higher optical loss due to higher N–H bonds absorption as well as lower density and higher roughness. Recently, reactive sputtering with DC-pulsed source using a silicon target under Ar/N_2_ plasma environment at temperatures below 100 °C was developed which provides another promising way to deposited Si_3_N_4_ film at low temperature [53]. Similar to silicon, numerous structures such as waveguides, ring resonators, photonic crystals, grating couplers, inverse tapers, Y-junction splitters, and multimode interferometers have been demonstrated using Si_3_N_4_. The usage of Si_3_N_4_ as a slab waveguide can date back to 1970s [54, 55]. With advances in technology, various optimization methods to reduce the losses of Si_3_N_4_ waveguides have been developed, such as high-temperature thermal annealing, chemical mechanical polishing, improved plasma etching, multipass lithography, photonic damascene process, and so on. Taking the waveguide loss values around 1.55 µm to better illustrate the development, in 2005, a low loss down to 0.1 dB/cm was reported by using a waveguide with a height of 150 nm and a width of 800 nm [56]. In 2014, a loss less than 0.01 dB/cm was reported using a highly delocalized resonator with a height of 40 nm, a width of 11 µm and a bending radius of 9.65 mm [57]. In 2016, a low propagation loss of ∼0.021 dB/cm was achieved in a “finger-shaped” Si_3_N_4_ resonator with an intrinsic Q around 17 million [58]. In 2017, an ultralow loss (<0.01 dB/cm) was achieved using a high confinement resonator with a height of 730 nm, a width of 2.5 µm and a bending radius of 115 µm [50]. Similarly, the photonic Damascene process has been used to fabricate very thick silicon nitride films (0.8–1.75 µm) hosting waveguides with a loss <0.05 dB/m, sometimes combined with a reflow process [59, 60]. Nowadays, Si_3_N_4_ has been used in numerous fields including integrated frequency comb generation, communications, spectroscopy, light detection and ranging, optogenetics, biosensing, optical coherence tomography, photonics quantum circuits, on-chip delay lines, atomic clocks, optical phased arrays, and narrow linewidth lasers [52, 61], [62], [63], [64], [65], [66], [67], [68], [69], [70], [71], [72], [73], [74], [75], [76], [77], [78], [79], [80], [81], [82], [83], [84], [85], [86].

#### 2.1.3 Silica

Silica material has a bandgap of 9.3 eV, covering a wide transparency range from visible to mid-IR (∼200 nm–3.5 μm). Silica-on-silicon optical waveguides are one of the most popular choices for photonic integrated circuits due to the mature fabrication process, low cost, low propagation loss, and refractive index contrast matching with optical fibers [18]. A large number of silica waveguide devices have been used in commercial optical equipment owing to their excellent optical performance, such as 1 × N optical splitters and AWGs. Due to the low index contrast between the silica core layer and the cladding layer, extremely low propagation loss of 0.08 dB/m in the silica waveguide was demonstrated [87] and a chemically etched ultrahigh-Q wedge-resonator on a silicon chip [88] is shown in Figure 2(c). However, due to the low refractive index of silica (∼1.44), the bending radius typically needs to be tens of millimeters, which leads to large footprints of the silica waveguide devices that are not suitable for large-scale, high-density, and compact photonic integrated circuits [89].

### 2.2 Non-group IV materials on silicon

Emerging applications will drive the demand for photonic integration platforms, which support not only the near-IR window, but also the visible and mid-IR operation, and drive the silicon photonic integration from low power and passive oriented components toward the next generation of active, high-power handling, and high-nonlinearity components. Looking for new materials with wider transparency windows, stronger electro-optic and nonlinear properties, and capability of supporting high-bandwidth modulation is inexorable for ultrahigh speed, energy-efficient communications and beyond. Additionally, materials that can be easily integrated with silicon photonics are attractive in terms of scalability and wide adoption. Some promising non-group IV materials and waveguides on silicon are the metal oxides (Al_2_O_3_, Ta_2_O_3_), direct bandgap materials (AIN and GaN), ferroelectric oxides (LN, BTO and PZT), and 2D materials. Table 2 summarizes the optical properties of the typical wideband materials which can be heterogeneously integrated on silicon.

**Table 2:** Optical properties of typical wideband materials heterogeneously integrated on silicon platform.

Parameter	Al_2_O_3_	Ta_2_O_5_	AIN	GaN	LN	BaTiO_3_	PZT
Experimental bandgap (eV)	5.1–7.6 [90]	4.4	6.0 (0.21 μm)	3.4	3.7 (0.34 μm)	3.2–3.3	3.35–3.89
Mean refractive Index(*n*) [*λ* = 632 nm]	1.77	2.13	2.16	2.38	2.29	2.41	2.40
To coefficient (K^−1^)	2.75 × 10^−5^	8.8 × 10^−6^ [91]	2.32 × 10^−5^	5.0 × 10^−5^ [92, 93]	3.34 × 10^−5^		
Kerr coefficient (m^2^ W^−1^)		6.2 ± 2.3 × 10^−19^ [91]	2.3 × 10^−19^	3.4 × 10^−18^ [94, 95]	1.8 × 10^−19^		8.5 × 10^−18^
Pockels effect (pm/V)			0.67 (*r*_13_) −0.59 (*r*_33_)	1.91 ± 0.35 (*r*_33_) 0.57 ± 0.11 (*r*_31_)	31.8 (*r*_33_)	1640 (*r*_42_)	−157.1 (*r*_33_)
Typical waveguide loss (dB/cm) [*λ* = 1.55 μm]	3 [*λ* = 371 nm]	4.8–5.4	0.8			10.5 ± 0.2 (TE), 9.0 ± 0.4 (TM) [96]	1

#### 2.2.1 Metal oxide (aluminum oxide (Al_2_O_3_, or alumina) and tantalum pentoxide (Ta_2_O_5_, or tantala))

**Figure 3:**
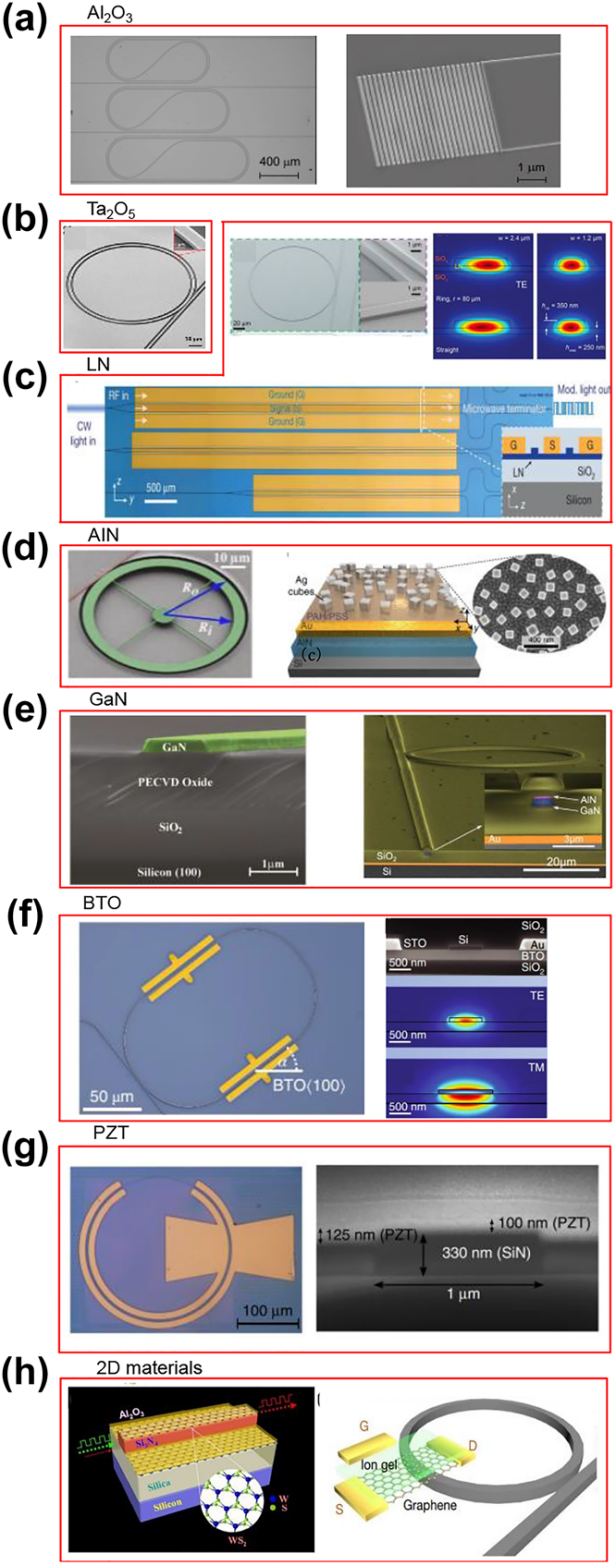
Representative devices of non-group-IV materials on silicon platform. (a) AI_2_O_3_: the SEMs of alumina straight, bent waveguides and fully-etched grating couplers [90]. (b) Ta_2_O_5_: a tantala resonator [91]. (c) LN: an ultrahigh Q LN microring resonator [109]; a CMOS compatible nanophotonic LN high speed modulator [112]. (d) AIN: an AlN suspended optomechanical microring resonator [126]; an AIN metasurface-pyroelectric detector [131]. (e) GaN: a cross-session SEM of GaN waveguide [141]; a GaN-on-insulator ring resonator on silicon [151]. (f) BTO: a BTO ring resonator with its cross-session SEM and TE/TM mode profiles [96]. (g) PZT: a PZT-on-SiN ring modulator [161]. (h) 2D materials: a schematic of the WS_2_-based optical modulator structure [178]; a conceptual design of the gate-tunable graphene–nitride heterogeneous microcavity Kerr frequency combs [165]. [(a) Reproduced with permission from reference [90]. Copyright © 2020, society of photo-optical instrumentation engineers; (b) reproduced with permission from reference [91]. Copyright © 2021, optical society of America; (c) reproduced with permission from reference [109]. Copyright © 2017, optical society of America; reproduced with permission from reference [112]. Copyright © 2018, nature publishing group; (d) AIN: reproduced with permission from reference [126]. Copyright ©2012 American institute of physics. Reproduced with permission from reference [131]. Copyright © 2020, nature publishing group; (e) reproduced with permission from reference [141]. Copyright © 2021, optical society of America; reproduced with permission from reference [151]. Copyright © 2022, optical society of America; (f) reproduced with permission from reference [96]. Copyright © 2019, nature publishing group. (g) Reproduced with permission from reference [161]. Copyright © 2018, nature publishing group. (h) Reproduced with permission from reference [178]. Copyright © 2018, American chemical society; reproduced with permission from reference [165]. Copyright © 2018, nature publishing group].

When the operating wavelength in the visible band or below is desired, metal oxides including aluminum oxide (Al_2_O_3_, or alumina) and tantalum pentoxide (Ta_2_O_5_, or tantala) are widely explored. Al_2_O_3_ has a wide bandgap from 5.1 to 7.6 eV [90]. Thanks to atomic layer deposition (ALD) and sputter deposition [97], Al_2_O_3_ waveguides show great potential for low loss integration from the ultraviolet (UV) to visible (208 nm–633 nm) [98]. Figure 3(a) shows the representative devices of alumina on silicon platform with straight, bent waveguides, and fully-etched vertical grating couplers, in which a waveguide loss of <3 dB/cm at a wavelength of 371 nm and a ring resonator with an intrinsic quality factor (Q factor) >4.7 × 10^5^ at a wavelength of 405 nm have been demonstrated [90]. Ta_2_O_5_ has a theoretical bandgap of 4.4 eV with a transparency window close to 250 nm. The unique property of Ta_2_O_5_ is the low but sufficient refractive index contrast to an oxide cladding at UV wavelengths, so that the sidewall scattering losses can be suppressed due to the diluted optical modes, and the measured waveguide losses below 0.3 dB/cm at wavelengths close to 633 nm [91] (Figure 3(b)) and ∼0.03 dB/cm across the entire telecommunications C-band [99] have been demonstrated respectively. Now Ta_2_O_5_ is emerging as a strong competitor to Si_3_N_4_ waveguide in terms of high refractive index, large bandgap, low stress, low optical loss, low thermo-optic coefficient, and 3-fold higher nonlinearity. During the fabrication of oxide cladded Ta_2_O_5_ waveguides, Ta_2_O_5_ can be deposited to make thick cores (e.g. 800 nm–1 μm) without the stress cracking issues of stochiometric Si_3_N_4_. Due to crystallization of the material, the annealing temperature of Ta_2_O_5_ is limited to approximately 600 °C, far below ∼1200 °C of Si_3_N_4_ annealing temperature needed to drive out hydrogen to achieve ultralow loss. A lower waveguide loss at 1.55 µm in Ta_2_O_5_ waveguide (core thickness ∼90 nm) has been demonstrated compared to that of Si_3_N_4_ waveguide with an equivalent core thickness [99]. Ring resonator structure with an intrinsic *Q* value of 3.8 million at 633 nm has been reported [91]. Additionally, Ta_2_O_5_ has a higher optical nonlinear coefficient (*χ*^(3)^ = 2 × 10^−13^ esu) [100], making it an attractive material for ultracompact and high optical confinement structures with wideband nonlinear applications including optical frequency combs and supercontinuum generation. Four-wave mixing experiments with high-confinement Ta_2_O_5_ waveguides and a relatively low optical loss of 1.5 dB/cm have been reported [101]. Al_2_O_3_ and Ta_2_O_5_ have also been actively studied in emerging applications, such as nanophotonic waveguide enhanced Raman spectroscopy (NWERS), in which the high index contrast waveguide excitation with visible light is critical [102]. The NWERS is a sensing technique involving the generation and detection of low levels of light, in which the level of background Raman autofluorescence from molecules in close vicinity of the waveguide is low compared to the desired Raman signal. Ahluwalia et al. [103] have compared both the Ta_2_O_5_ and Si_3_N_4_ waveguide Raman autofluorescence background with respect to their own background with excitation in the range 488–640 nm in direct stochastic optical reconstruction microscopy (dSTORM) for single molecule localization. At 640 nm, a comparable excitation background was observed between the two materials. However, at shorter wavelengths, Ta_2_O_5_ is expected to perform better as the background for Si_3_N_4_ significantly increases, which reduces the localization precision for dSTORM imaging.

#### 2.2.2 Lithium niobate

Lithium niobate (LiNbO_3_ or LN) has a wide transparency window from UV to mid-IR and low absorption loss. It has been the most successful optical material for commercial modulators due to its large nonlinear and electro-optic coefficients (non-centrosymmetric crystal with strong Pockels effect). LN modulators have been commercially available for decades along with the great success of optical fiber communications that underpin the internet [104]. The recent revolution in the LN photonic industry has been sparked by the thin-film lithium niobate (TFLN) waveguide with performance far exceeding the traditional bulk lithium niobate devices [104], especially with the innovations of nano-fabrication of LN-on-insulator (LNOI). With all these good optical performances in the telecommunication band, LNOI opens an avenue to outperform bulky LN devices in footprint and cost, and realize integrated on-chip photonic devices with unprecedented performances in terms of propagation loss, optical nonlinearity, and electro-optic tunability. A typical LNOI wafer (3 or 4 inches) generally has a sub-micrometer LN film thickness (∼300–900 nm) and is suitable for mass production of nanostructure building blocks with ridge waveguide fabrication techniques. For the rib waveguides etched into the LN thin film layer and bonded on the buried silicon dioxide layer, the index contrast can vary between 0.7 and 1 with or without silica superstrate [105], offering benefits of compact mode size with low bending loss and small radius, high efficiency for electro-optic interaction, and nonlinear optical process with strong modal confinement [53, 106]. The TFLN based ridge waveguide by dry etching has shown a low loss of 0.3 dB/cm at 1.55 µm [107]. Furthermore, for waveguides fabricated by direct etching and by using CMP together, the surface roughness of LNOI nanostructures is around 0.1 nm [108]. A LN microring resonator with a record low loss of 0.027 dB/cm and ultrahigh Q factor of 10^7^ [109] (Figure 3(c): left) and a resonator with a high Q of 11 million [110] have been demonstrated. A LNOI based modulator can also achieve high bandwidth, low drive voltage, low insertion loss, and compact size. Especially, compared with the value of 10–20 V cm of typical bulk LN modulators, a half-wave voltage-length product *V*_*π*_ *L* of 2–3 V cm [111] has been demonstrated. A typical nanophotonic LN modulator compatible with CMOS drive voltage of *V*_*π*_ = 1.4 V and 3-dB electro-optic bandwidth up 100 GHz [112] is shown in Figure 3(c): right. Although lithium niobate has a wide transparency window ranging from UV to mid-IR and great progress has been made along the development of high-performance devices at telecom wavelengths in near-IR band, less work has been demonstrated for other wavelength ranges [106, 113]. Recently, an ultralow-loss integrated visible photonics platform based on thin-film LNOI has been demonstrated [110]. These demonstrated waveguides feature an ultralow propagation loss of 6 dB/m and the microring resonators feature a high intrinsic Q of 11 million at 637 nm wavelength. An on-chip visible light intensity modulator with an electro-optic bandwidth of 10 GHz was also demonstrated. The visible or mid-IR light in LNOI can also be generated by using wavelength up-conversion or down-conversion in telecom wavelengths as it possesses both large *χ*^(2)^ and *χ*^(3)^ nonlinearities and a wide transparency window from 0.3 to 5 µm. For example, the broadest supercontinuum generation (SCG) in a monolithic dispersion-engineered LN waveguide, spanning from 0.35 to 4.1 μm at a pulse energy of 240 pJ has been demonstrated [114]. The visible spectrum from 0.45 to 0.8 µm through second harmonic generation (SHG) and sum frequency generation (SFG), the UV spectra at 360 nm through cascaded SHG via phase-matching to higher spatial mode, and mid-IR combs from 3 to 4 µm via difference frequency generation (DFG) were observed. The near-UV light may be further generated based on Mg-doped LN. Ultralow-loss LN waveguides with strong second-order and third-order nonlinearities, together with the integration of on-chip light sources, may open up opportunities for various novel devices for frequency metrology, sensing and quantum information processing in UV, visible, and mid-IR regimes.

#### 2.2.3 Aluminum nitride

Aluminum nitride (AlN) is another type of wideband semiconductor material that can be integrated on silicon platform. AlN has a large bandgap of 6.2 eV, a wide transparency window covering from UV to mid-IR, and a significant second-order nonlinear optical property contributed by its non-centrosymmetric crystal structure. AlN is a ceramic with low dielectric constant and excellent mechanical properties. It is non-toxic and has a linear expansion coefficient similar to that of silicon. In addition, it has high thermal conductivity and excellent electrical insulation properties, making it an ideal material for many electronic applications. Furthermore, it also exhibits piezoelectric and pyroelectric effects, which enable optomechanical devices and pyroelectric photodetectors. AlN on silicon has been widely used for sensors, actuators [115, 116], and also successfully demonstrated for UV LEDs [5]. Various standard functional devices such as couplers, waveguides, ring resonators, optomechanical devices, emitters, photodetectors, and metasurfaces have been demonstrated. In 2012, AlN-on-insulator has been proposed as a new material system for integrated optics by Pernice et al. [117]. AlN thin films were sputtered onto a 2.6 µm thick SiO_2_ on silicon substrate. The AlN film is highly c-axis oriented with a rocking curve full-width at half-maximum of 2° at AlN’s [0002] peak. The film thickness is chosen to be 330 nm on this platform and fabricated device propagation loss down to 0.8 dB/cm is reported at 1.55 µm. In 2014, the working wavelength of an AlN waveguide has been extended to the mid-IR wavelength regime (*λ* = 2.5 μm) with a propagation loss of 0.83 dB/cm [118]. In 2019, by leveraging the same sputtered AlN on SiO_2_ platform, the working wavelength of the photonics devices has been further extended beyond 3 μm [119]. The electro-optic coefficient of AlN has been reported to be *r*_13_ = 0.67 pm/V, *r*_33_ = −0.59 pm/V (measured at 633 nm) [120], which despite being significantly less than LN, could still be beneficial due to its ease of film deposition. Such an electro-optic effect enables the demonstration of phase shifters and optical modulators using AlN [121, 122].

Compared with thermo-optic-effect-based phase shifters, the electro-optic effect has the advantage of low power consumption and fast tuning speed. The electrodes are placed at the top and the bottom of the AlN waveguide. The maximum electric field created by the voltages applied on electrodes can go through the AlN waveguide. The modulation efficiency (*V*_*π*_⋅*L*_*π*_ product) measured from the fabricated waveguide-ring resonators and MZI modulators near the 1.55 µm wavelength is ∼240 V cm for the transverse electric (TE) mode and ∼320 V cm for the transverse magnetic (TM) mode. Currently, the modulation speed is estimated to be 30 MHz which is limited by the speed of the voltage supplier. Besides, the modulation efficiency of the present AlN phase shifter is low due to the relatively small Pockels coefficient of AlN. The piezoelectric coefficient of AlN thin film has been reported to be *d*_33_ = 5.53 pm/V, *d*_31_ = −2.65 pm/V [123, 124]. Such piezoelectric property enables the wide utilization of AlN in MEMS [115, 116]. The piezoelectric property together with optical properties of AlN discussed earlier, make AlN a suitable material for optomechanical devices. A number of MEMS-based optomechanical devices have been demonstrated [125, 126]. Also, contributed by the piezoelectric property and photo-elastic constant (*p*_13_ = −0.019, *p*_33_ = −0.107 [127]), AlN has become a platform to investigate the photon–phonon interaction within solids [128]. Figure 3(d): left shows an optical micrograph of a suspended AlN ring resonator made adjacent to a straight coupling waveguide. A loaded Q of 125,000 is measured and three mechanical modes are found at 30.6 MHz, 47.3 MHz, and 1.04 GHz for this fabricated optomechanical device. The GHz AlN optomechanical resonators demonstrated in Ref. [119] can find applications in precision oscillators and high-speed ultrasensitive systems, as well as in acoustic nonreciprocal devices such as isolators. The pyroelectric coefficient of AlN has been reported to be 0.0033 μC/(cm^2^ K) [129]. Such pyroelectric effect enables the realization of pyroelectric photodetector using AlN [130–132]. By integrating a plasmonic metasurface with an aluminium nitride pyroelectric thin film on Si substrate (schematic shown in Figure 3(d): right), spectrally selective, room-temperature pyroelectric detectors from 0.66 to 2 µm with an instrument-limited 1.7 ns full width at half maximum and 700 ps rise time are demonstrated. Heat generated from light absorption diffuses through the subwavelength absorber into the pyroelectric film producing responsivities up to 0.18 V W^−1^ due to the temperature-dependent spontaneous polarization of the pyroelectric films.

A significant second order nonlinear effect for AlN thin film has been reported on either sapphire or Si substrate. However, the second harmonic generation efficiency and *χ*^(2)^-based optical parametric generation are severely limited by the intrinsic propagation losses. Single-crystalline AlN epitaxially grown on sapphire substrate has demonstrated much lower loss compared with the polycrystalline AlN-based cavity [133, 134]. As a result, most of the nonlinear studies are conducted with the epitaxially growth single-crystalline high-quality AlN on sapphire substrate rather than using sputtering AlN on Si substrate, and it will not be discussed in detail here.

#### 2.2.4 Gallium nitride

III-N family compounds such as GaN and its alloys – AlGaN and InGaN, are other promising candidates for integration on the silicon platform. They are favorable for light emitting devices (LEDs, LDs) [135, 136] due to their direct bandgap property, and their emission spans a wide range – from the UV to the visible and a wide transparency range from the UV to mid-IR [137]. GaN exhibits high breakdown voltage (>3 MV/cm) [138], moderate thermo-optic coefficient (∼5 × 10^−5^) [92, 93] and resilience to harsh environments – including elevated temperature, corrosive environment and ionizing radiation [139]. The ability to grow multilayer structures consisting of AlGaN and InGaN alloys enables high flexibility in band structure design as well as in finely tailoring the grown structure’s refractive indices. Due to its wurzite non-centrosymetric structure, GaN also possesses 2nd order nonlinearity. Its *χ*^(2)^ coefficient was measured in bulk platform and in a waveguide configuration and values of around 10 pm/V were reported [140, 141]. Its third-order nonlinear coefficient is about an order of magnitude higher than that of Si_3_N_4_, LN, and AlN (*n*_2_ = 3.4 × 10^−18^ m^2^/W [94, 95]), leading to exciting opportunities in nonlinear photonics applications. GaN is epitaxially grown mostly using MOCVD, but there are some niche applications requiring MBE growth. The control of its high-quality epitaxial growth, doping and fabrication processes are in wide usage and are gaining maturity. The Nobel prize in physics in 2015 was awarded on the development of the GaN based blue LED [142]. It has led to a well-established industry and is the second most processed semiconductor after Si. Therefore, photonics based on GaN is appealing not only because of its unique characteristics, but also due to its solid industrial potential.

In order to integrate GaN on a Si substrate, direct growth is possible in principle with the aid of strain relief buffer layers [143, 144]. However, due to the lower RI in GaN than Si – 2.3 compared to 3.7 at 1.55 µm, a few micron of SiO_2_ is necessary between the layers as an optical isolating layer. Therefore, wafer bonding approach is preferred, resulting in a GaN on insulator (GaNOI) on silicon platform.

Many passive photonic devices have been demonstrated on GaN platform, including low loss waveguides [95, 145], directional couplers [146], frequency converters [141, 147] and more [148]. Waveguides and resonators with propagation losses as low as 1 dB/cm [145] and 0.26 dB/cm [149] (respectively) were reported at telecom wavelengths. In the visible range, loss values of ∼2 dB/cm were reported [150]. On-chip nonlinear optical devices were demonstrated, utilizing the wide bandgap of GaN and the fact that TPA is negligible for wavelengths above 730 nm. The typical GaN devices of waveguide and resonator are shown in Figure 3(e), and second harmonic generation from 1.56 µm to 780 nm has been demonstrated by Xiong et al. [141] and by Gromovyi et al. [151] on GaNOI platform, respectively. GaN electro-optic coefficient (Pockels) has been reported to be *r*_13_ = 0.5 pm/V, *r*_33_ = 1.9 pm/V [152]. Few theoretical works were done in the design of active devices such as modulators and phase shifters [153, 154]. However, unlike AlN, such devices have not been experimentally demonstrated yet.

#### 2.2.5 BTO

Barium titanate (BTO) has a bandgap of 3.2–3.3 eV. It has garnered substantial interest in the past few years due to the very high $\chi^{\left(2\right)}$ , which leads to a large bulk Pockels coefficient >1600 pm/V, approximately 50 times higher than LN. However, previous attempts to integrate them with MgO waveguides degraded their effective $\chi^{\left(2\right)}$ by one or more orders of magnitude [155], or were limited to slow speeds <1 MHz [156]. This is partially because that the $\chi^{\left(2\right)}$ depends on the non-centrosymmetric crystalline structure of the material, and this effective non-centrosymmetry can be reduced by random orientations of domains formed during growth by pulsed layer deposition, molecular beam epitaxy (MBE) or metal-organic CVD (MOCVD). While photonic crystal structures have achieved higher modulation bandwidths of 4.5 GHz using 500-nm thick MOCVD films of BTO, they were restricted to MgO substrates [157]. Recent work has used MBE-grown BTO films wafer bonded to silicon to overcome these challenges, leading to large Pockels coefficients >900 pm/V [96]. The BTO ring resonator with its cross-sessional SEM and TE/TM mode profiles are shown in Figure 3(f). The films had a substantial fraction of the optical field (∼40%) in just 50 nm thin BTO films despite its lower index than silicon. All these improvements led to a high analog modulation bandwidth of 30 GHz. The current state-of-the-art loss is about 6–10 dB/cm [158]. Even though the absorption edge of BTO is reported around 300 nm, due to the high losses, the number of reported works about BTO devices on silicon substrates are very limited. As progress in BTO-Si devices has demonstrated cryogenic operation [159] and non-volatile memory implementations [160], future developments would need to reduce the propagation losses significantly.

#### 2.2.6 PZT

Piezoelectric lead zirconate titanate (PZT) has a bandgap ranging from 3.35 to 3.89 eV and it is another type of material with large Pockels effect. Its Pockels effect is several times higher than that of LN. PZT thin films also show propagation losses down to 1 dB/cm and have been successfully integrated with silicon nitride films to demonstrate Pockels modulators with a 33-GHz analog bandwidth [161], as shown in Figure 3(g). The lower loss is achievable both due to the PZT material itself as well as the lower absorption and scattering loss in the silicon nitride substrate. Previous works have shown phase modulation at sub-MHz speeds using PZT-on-silicon nitride films but with significantly lower sub-MHz bandwidths, although wafer scale foundry compatible fabrication has been demonstrated in some of these works. Another attractive feature of PZT films is the ease of direct deposition on nearly any substrate through spin-coating and annealing (also called sol–gel), which is compatible with CMOS front-of-the-line processes [161]. This is simpler than wafer bonding of crystalline or epitaxially grown films that is needed for other $\chi^{\left(2\right)}$ materials such as lithium niobate or barium titanate. Future improvements could look at further reducing the losses, increasing modulation bandwidths and effective Pockels coefficients (currently at ∼150–250 pm/V) [162] through optimized waveguide design, placement of electrodes, or modifications to the stoichiometry of the material by incorporating suitable lanthanides [163].

#### 2.2.7 2D materials

Another main development of wideband silicon photonics is the integration of two-dimensional (2D) materials on the silicon platform. 2D materials, including graphene, black phosphorus (BP), and tungsten disulfide (WS_2_), etc., are the 3D crystals in the form of a planar structure regarded as with a negligible thickness in one dimension. 2D materials have shown fascinating optoelectronic properties in windows ranging from visible to mid-IR due to their rich band structures from 0 to several eV [164], and demonstrated attractive optical properties that enable the generation, manipulation, and detection of light [164]. Thanks to their unique atomic flexibility, various 2D materials are conveniently integrated on silicon-based platforms without significantly affecting the mode fields, either on-chip [165] or on-fiber [166]. Of which, graphene is a single atom thick carbon sheet with atoms arranged in a hexagonal structure, and was first and most widely studied for integrated optoelectronic devices due to its excellent optical and electrical properties [167]. A mono-graphene layer has a wide transparency wavelength ranging from infrared to visible due to a constant absorption of 2.3%. It also has a high thermal conductivity (5300 W m^−1^ K^−1^) and a high carrier mobility of 2 × 10^5^ cm^2^ V^−1^ s^−1^ at room temperature with electrical-controllable permittivity. Additionally, graphene has a large Kerr coefficient (*n*_2_ = 10^−12^ m^2^ W^−2^) which is highly favorable in nonlinear photonics [164]. The combination of 2D materials and silicon photonics has been bringing unprecedented new opportunities for silicon-based photonic devices, from lasers, modulators, photodetectors to nonlinear devices. 2D material based lasers on silicon platforms have been realized and mainly include two categories, one is the saturable absorption effect of 2D materials to achieve Q switched or mode-locked pulse output [168], and the other one uses 2D materials as gain media for actuation [169]. As saturable absorbers (SAs), 2D materials have achieved mode-locking of different lasers, covering different wavelengths from visible to mid-IR (500 nm–2.5 µm) [170]. For instance, Bharathan et al. report on the feasibility of MXene and platinum diselenide (PtSe_2_) as novel saturable absorbers for the development of wavelength stabilized passively mode-locked mid-infrared fiber laser systems [171]. Besides, laser excitation based on WSe_2_ [172], WS_2_ [173], MoTe_2_ [174] and MoS_2_/WSe_2_ heterostructure [175] have also been achieved within various silicon-based microcavities. 2D material based silicon modulators demonstrate ultrafast and broadband responses [176]. For instance, by integrating graphene heterostructures in silicon nitride microrings, Phare et al. achieved an electrically tuned resonance spectral shift, leading to ultrafast electro-optic modulation with a 30-GHz bandwidth [177]. More recently, Yang et al. integrated WS_2_ with a silicon nitride waveguide and successfully achieved all-optical modulation and amplification of optical signals at 640 nm by modulating a 532 nm pump light source [178], as shown in Figure 3(h): left. Here, WS_2_ can be used as a gain medium, which theoretically incurs lower loss and exhibits higher contrast level and signal-to-noise ratio. To date, versatile 2D materials-based modulators including different materials (e.g. graphene, TMDs, BPs, and other types) and different modulation mechanisms (e.g. optical, electrical, and thermal) cover the visible, infrared, and terahertz ranges [179, 180]. Photodetectors based on 2D materials was firstly reported by Xia et al. in 2009 [181]. Compared to traditional semiconductor photodetectors, 2D materials based photodetectors demonstrate appealing advantage in terms of bandwidth, possibly exceeding 500 GHz [182]. In 2D material photodetectors, the absorbed optical signals can be converted into electrical signals through a variety of physical mechanisms [183], such as the photothermalelectric effect (PTE), the photo-bolometric effect (PBE), the photoconductive effect (PCE) and the photovoltaic effect (PVE). In 2018, by interconnecting quasi-one-dimensional nanoribbons to form plasmonic resonator arrays, Guo et al. demonstrated a mid-infrared graphene detector, enabling the electrical detection of plasmon decay in the nearby graphene resonators with an external responsivity of 16 mA W^−1^ and a low noise-equivalent power of 1.3 nW Hz^−1/2^ at 12.2 μm [184]. Later, Liang et al. reported a high-performance ultra-wideband photodetector based on PdSe2 with a unique pentagonal atomic structure, obtaining response from visible to mid-infrared range (up to ≈4.05 µm). The device stably operates in ambient and at room temperature, with a responsivity of 708 A/W at the wavelength of 1.064 µm [185] due to the photogating effect in 2D materials. Recently, Wu et al. designed a vertically stacked back-to-back 2D/3D hybrid photodetector using a black phosphorus (BPs)/molybdenum sulfide (MoS2)/silicon (Si) vdWs heterostructure in temporal-spatial coexisting near-IR/mid-IR two-color blackbody sensitive photodetectors, and showed ultralow crosstalk of ∼0.05% at room temperature [186]. 2D materials can also help to break the limitation that the nonlinear response in conventional integrated photonics is typically low. In 2018, using a graphene incorporated microring, Yao et al. reported controllably diverse gate-tunable graphene–nitride heterogeneous microcavity Kerr frequency combs in one single device [165], a conceptual design is shown in Figure 3(h): right. Furthermore, by integrating graphene in different optical paths (e.g. waveguides [187], integrated D-shaped fibres [188, 189] and silica microspheres [9]), researchers also accomplished the modulation of the second-order, third-order nonlinearity and group index of graphene, achieving tunable plasmonic generators and high performance sensors. Recently, via depositing a monolayer of crystalline Mo_3_Te_4_ in a 1-THz-FSR Si_3_N_4_ microcavity, He et al. demonstrated a 2D material enhanced microcavity that can have a third order nonlinear susceptibility *χ*^(3)^ = 10^−18^ ∼ 10^−15^ esu, potentially useful to ultralow threshold frequency conversion applications [190]. It can be seen that the 2D materials will become more attractive over the next few years covering a wideband as the fabrication maturity and control of the material properties increase rapidly.

To conclude this part, the integration of wideband material on silicon platform can enable new functionalities and play a critical role in the integration of light source, detectors, and the operation of devices not only near-IR but covering the visible and mid-IR range. Figure 3 shows the typical optoelectronic devices based on non-group-IV family materials on silicon platform. Yet, there are a relatively low number activities and reports on this platform, leaving room for further research with great potential applications.

### 2.3 General fabrication method and low loss process treatment

**Figure 4:**
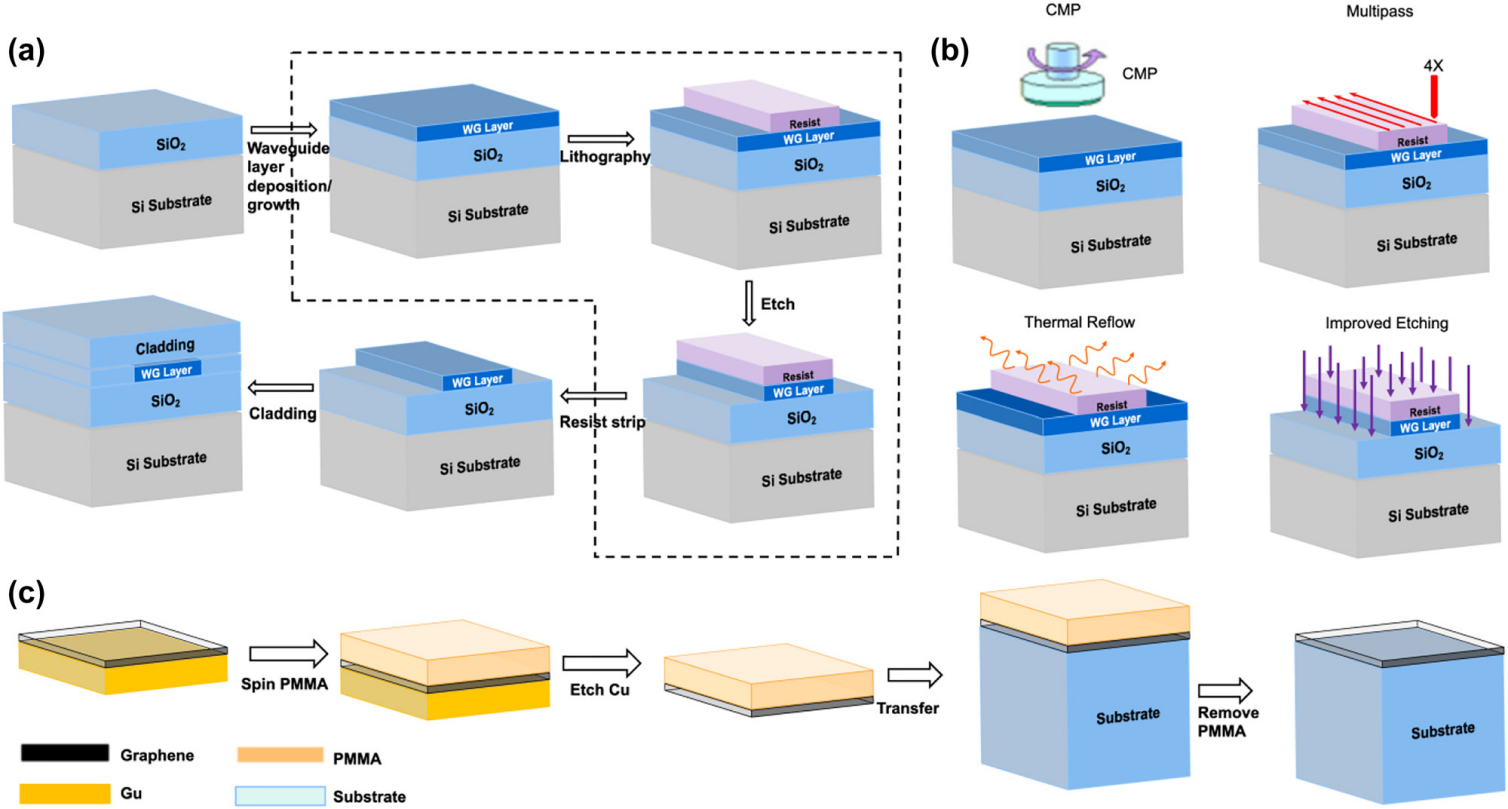
General fabrication method and low loss process treatment. (a) General fabrication method. (b) Low loss process treatment. (c) The fabrication process for 2D material integrated optoelectronic devices on a silicon platform based on the wet-transfer technique.

The general fabrication method and low loss process treatment of different optical materials on the silicon platform are illustrated in Figure 4(a). For these deposited thin film waveguide-based materials on insulator wafer (silicon, Si_3_N_4_, AlN, GaN, Al_2_O_3_, LN, BaTiO_3,_ and PZT), they generally follow the same fabrication process. First, a waveguide layer is deposited on to the silicon substrate; SiO_2_ is the most commonly used insulating material on silicon due to its relatively low refractive index and ease to fabricate. Then the waveguide layer is patterned using lithography, such as e-beam lithography (EBL), deep-ultraviolet (DUV) lithography, and contact lithography. After exposure and the resist is developed, the design pattern is ready for transfer. Etch is used to transfer the pattern from the resist to the underlying waveguide layer. Depending on the materials and the ultimate goals, it can be dry etch or wet etch. Dry etch typically etches quickly and anisotropically, it is particularly useful for etching chemically resistant materials. After successfully transferring the patterns, the leftover resist is stripped, and cladding material is deposited to protect the waveguide layer. At this stage, the wafer is ready for dicing or further processing such as heater deposition, metal pad deposition, heterogenous integration, etc. Low-loss waveguide is the key to achieve high-performance integrated optoelectronic devices. Waveguide layer deposition, lithography and etch are the three key process steps to determine the loss. Many groups have been working on these steps to reduce the devices’ losses. We have listed a few methods that can help to achieve low loss as shown in Figure 4(b). As an effective process, chemical mechanical polishing (CMP) is widely used to reduce thin film surface roughness, thus reducing scattering. In addition, during the LN fabrication process, CMP has also been used to polish the sidewalls of the waveguide to reduce the scattering loss. In this process, a chromium layer is used as the CMP mask, and the pattern is formed by femtosecond laser ablation to define the shape of the LN waveguide. Then, by using a wafer polishing machine, the exposed LN film can be removed to form the waveguide. Finally, the chromium layer at the top of the LN waveguide can be removed by wet etch using HF solution. The sidewall angle of the LN waveguide can be adjusted from a few degrees to nearly 80° by controlling the duration of CMP process [191]. A second CMP process can be performed if the upper surface of the waveguide is very smooth, a root-mean-square surface roughness as low as 0.452 nm, and a low loss of 0.1 dB/cm has been demonstrated [108]. Other methods have been developed to reduce losses, such as multi-pass lithography to reduce line-edge roughness by exposing the same pattern multiple times, improved etch recipe to reduce polymer residue by optimizing the gas ratio, and resist reflow to provide smoother sidewalls by using tens of hours of high‐temperature annealing.

The fabrication process for 2D material integrated optoelectronic devices on a silicon platform is a special process and it is commonly based on the wet-transfer technique, as shown in Figure 4(c). Taking graphene as an example, a polymethyl methacrylate (PMMA) layer is spin-coated on the graphene during the wet-transfer process, and the copper foil is etched by a commercially available etchant (copper etchant Type CE-100, or a 0.1 m (NH_4_)_2_S_2_O_8_, or ferric chloride (FeCl_3_) solution). Then, the PMMA-graphene film is moved to distilled water to rinse the etchant residue. Subsequently, the film is transferred to the substrate, and the chip is dried overnight in air. Finally, the PMMA can be removed by acetone, and then the entire chip is covered with graphene layer.

**Figure 5:**
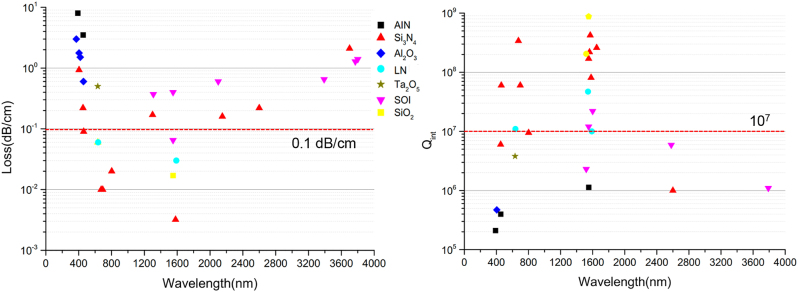
Summary of reprehensive waveguide losses and intrinsic Q factors of different optical materials on silicon platform across visible, near IR, and mid-IR spectra. (left) Waveguide loss of SiO_2_ [192], silicon and SOI [39, 194], [195], [196], [197], [198], [199, 202], Si_3_N_4_ [50, 57, 203], [204], [205], [206], [207], [208, 210], AlN [210], Al_2_O_3_ [90, 204], Ta_2_O_5_ [91], and LN [109, 110]. (right) Intrinsic *Q* values in different optical materials of SiO_2_ [88, 193], silicon and SOI [37,39, 200], [201], [202], Si_3_N_4_ [50, 57, 203, 205, 207, 209, 211], AlN [212, 213], Al_2_O_3_ [90, 214], Ta_2_O_5_ [91], and LN [109, 110, 215].

Following the successful model of SOI foundry, ultra-wideband integrated photonics on silicon platforms are highly desired that are compatible with cost-effective wafer-scale fashion for more emerging applications. They are most likely to support heterogeneous integration of multiple wide bandgap material systems. However, the waveguide loss sets a fundamental limit to the performances of the integrated devices. The loss of a waveguide mainly includes the absorption loss, radiation loss and scattering loss. While the regime of ultralow loss has been achieved at telecom band, progress at wideband spectrum covering visible and mid-IR wavelengths is limited, because SOI platform is almost opaque or suffers from large absorption loss. To date, there are only a few materials that are highly transparent over the entire UV-to-visible spectrum. In the short wavelength range down to visible, the Rayleigh scattering from the waveguide roughness due to the non-idealities of fabrication will inevitably induce loss, which contributes significantly at the lower end of the visible band when scaling with the wavelength *λ* (as 1/*λ*^4^). Therefore, it is necessary to use materials that can be fabricated with low roughness to develop low-loss integrated photonic devices in the visible range. In the long wavelength range up to mid-IR, due to the strong absorption of the buried oxide in the range between 2.6 µm and 2.9 µm and beyond 3.6 µm, the development of low-loss waveguide is also immature. To enable the advances in visible and mid-IR band, it is expected that the integrated solution should be CMOS compatible at wafer scale, capable of supporting various photonic components, with fundamental ultralow waveguide losses (<0.1 dB/cm), and ultrahigh Q resonators (>10^7^). Here, in Figure 5 we summarize the representative low-loss and high intrinsic Q factor of aforementioned silicon based material or wide bandgap waveguide core material choices in the visible, near-IR and mid-IR range, including SiO_2_ [88, 192, 193], silicon, and SOI [37, 39, 44, 194], [195], [196], [197], [198], [199], [200], [201], [202], Si_3_N_4_ [57, 203], [204], [205], [206], [207], [208], [209], [210], [211], AlN [212, 213], Al_2_O_3_ [90, 204, 214], Ta_2_O_5_ [91], and LN [109, 110, 215]. It can be seen that most of the impressive works still focus on the near-IR range. The waveguide loss and Q factors lag behind in the visible and mid-IR range. However, currently Si_3_N_4_ has been shown to be the most mature and versatile wide bandgap material covering visible and mid-IR bands with low propagation losses for the wavelengths ranging from 532 nm to 3.7 µm. Other non-group IV materials are still relatively undeveloped with fewer reports, but may excel in certain functions due to the unique properties aforementioned. Importantly, there are no one-fit-for-all solutions for high performance photonic devices from visible to mid-IR on silicon platform. The key factor to choose different wide bandgap materials for heterogenous integration include the optical properties (intrinsic material absorption in the core, cladding and substrate, nonlinearity and electro-optical properties, etc.) and the delicate waveguide design and fabrication process (optical confinement, waveguide side/top-wall scattering, etc.).

## 3 Typical visible, near-IR, and mid-IR applications

There is no doubt that telecommunication is the most successful application for silicon photonics. With the advantages of natural transparency in near-IR and CMOS compatibility, and spurred by the well-developed fiber as well as the erbium-doped fiber amplifier (EDFA) technologies, silicon photonics has started from its roots in telecommunication applications and has made remarkable development since its inception in the 1980s [216]. Numerous researches have been demonstrated in silicon photonics with various high-performance components in the telecommunication band [25, 35, 217, 218], i.e. waveguide, modulator, AWG, photodiodes, etc., and now reached sufficient maturity for commercial telecommunication applications at production volumes [26, 27, 35]. Now it has been extended to a plethora of application fields covering visible, near-IR, and mid-IR bands. However, silicon has well-known shortcomings in terms of limited wavelength range, lack of second-order optical nonlinearity, TPA and nonlinear absorption, etc. This promotes the integration of other aforementioned wideband materials on the silicon platform. Here we will focus on three typical wideband application scenarios as examples.

### 3.1 Integrated sensing and imaging technologies

Sensing is a field which greatly benefits from integrated optical components, especially photonic devices based on the silicon platform. These devices provide incomparable advantages such as high sensitivity, integration ability with electronic devices, compact footprint, low-cost, and semiconductor mass manufacturing compatibility. Waveguide-based devices and ring resonators are becoming more and more attractive in sensing applications such as chemical and biosensing. They are typically based on the variation of optical properties in the waveguide or ring resonator. When light propagates into an optical waveguide, a certain amount of power travels into the core, while the remainder is confined to the cladding and substrate regions which is called the evanescent field. In most cases, the evanescent field interacts with the analyte near the surface, and the effective index or absorption coefficient will be changed. As a result, various gases and chemical species can be detected. In other systems, the light is emitted or collected through photonic integrated circuits so that the phase and amplitude of the light and the delay introduced during its propagation can be measured. One example in bioimaging and health care is known as optical coherence tomography (OCT) which uses low-coherence light through fibers or photonic integrated circuits to capture the cross-sectional and three-dimensional images with micrometer resolution.

**Figure 6:**
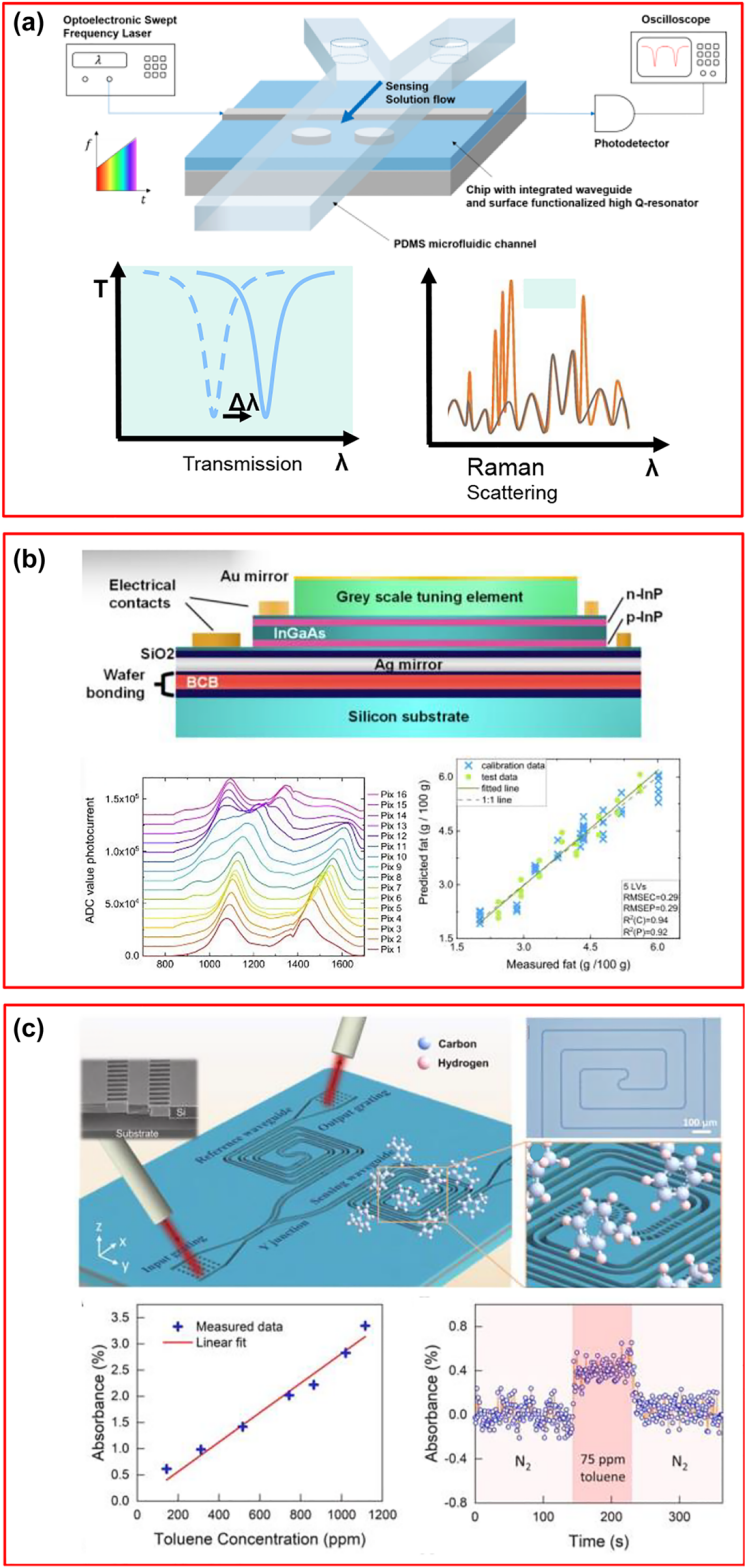
Typical integrated sensing devices. (a) Schematic of bio-sensing using optical micro-resonators [234]. (b) An array of resonant-cavity-enhanced photodetectors suitable for diffuse transmittance/reflectance measurements in the 850 nm–1.7 µm wavelength ranges on silicon substrate and the use of the multi-pixel array for the measurement of the nutritional properties of milk is presented [227]. (c) Example of mid-IR sensing based on the suspended silicon waveguide platform. Toluene vapor detection is presented with a corresponding limit of detection of 75 ppm. The response and recovery time to 75 ppm toluene are about 0.8 and 3.4 s, respectively [233]. [(a) Reproduced from website [234] http://www.its.caltech.edu/%7Eaphyariv/research.html; (b) reproduced with permission from reference [227]. Copyright © 2018, nature publishing group. (c) Reproduced with permission from reference [233]. Copyright © 2021, De Gruyter].

The visible spectral region is of particular interest for biosensing and imaging. However, silicon only offers good transmittance starting from 1.2 µm, and because of the relatively low refractive index of SiO_2_, Si_3_N_4_ becomes more attractive. Highly accurate sensors based on Si_3_N_4_ have been reported for chemical and biological samples, such as DNA biosensor, methane sensor, and fluorescence-based sensors [219–222]. Other than these sensors, many efforts have also been made to address the need for simplification and miniaturization of sensing system parts using photonic integration, such as absorption and hyperspectral microscopes, cytometers, and OCT systems [62, 223], [224], [225], [226]. Figure 6(a) shows the schematic of the bio-sensing using optical micro-resonators.

The near-IR spectral region is interesting for the sensing of organic materials due to the presence of absorption bands caused by overtones and combinations of vibrational modes of O–H, C–H, and N–H bonds. Near-IR can provide higher sensitivity than visible solutions for important application cases such as food science and health care. As an example, an array of resonant-cavity-enhanced photodetectors suitable for diffuse transmittance/reflectance measurements in the 850 nm–1.7 µm wavelength range is recently demonstrated on silicon substrate, as shown in Figure 6(b). The use of the multi-pixel array for the measurement of the nutritional properties of milk is presented [227]. In addition, near-IR is ideal for 3D imaging applications because it is invisible to the human eye, making it unnoticeable when cast into a user’s face, eyes, or environment. Applications such as eye-tracking, short-range and long-range automotive Lidar, and non-invasive detection for point-of-care are all relied on the near-IR wavelength range.

The mid-IR spectral region is known as the molecular-fingerprint region. Most molecules have strong fundamental vibrational bands there, thus providing a unique way to identify and quantify molecules. The strength of transitions in this spectral band may be more than a thousand-fold stronger than that in the near-IR telecom region, enhancing detection sensitivity by a similar proportion. Silicon photonics has long been considered unsuitable for mid-IR applications due to its lack of broad transparency beyond the near-IR range. The transparency window of the silicon waveguide core extends to 6–8 μm wavelength, whereas the SiO_2_ cladding material begins absorbing strongly around 3.5 μm. Therefore, few demonstrations of SOI waveguides have operated past 2.5 μm. To circumvent the limitation, efforts have been made to replace the lossy SiO_2_ cladding with other materials exemplified by silicon-on-nitride or with air cladding suspended silicon structures. With these efforts, the wavelength limit has been pushed beyond 3.5 μm [37, 45, 228], [229], [230], [231], [232] and these structures can be used for gas sensing. For example, sensing based on the suspended silicon waveguide platform is demonstrated in Figure 6(c) [233]. Another example is CO_2_ sensing. The absorption coefficient of the pure concentration CO_2_ is larger than 80 cm^−1^ at the mid-IR wavelength around 4.3 μm. This strong absorption gives the ability to achieve the high sensitivity sensing of CO_2_ with the mid-IR photonics. A CO_2_ gas sensing structure based on a silicon-on-nitride strip waveguide was developed and the achieved limit of detection was 5000 ppm CO_2_ at 4.23 μm [232].

### 3.2 Integrated optical frequency comb technologies

Optical frequency combs (OFCs) can offer an unrivalled degree of frequency measurement precision that underpins the advance of modern technology in both fundamental research and industry applications. By leveraging wide bandgap nonlinear optical materials, tightly-confined waveguide geometry and wafer-scale photonic foundries, the integrated OFC technology provides a novel route to realize compact, low-cost, and energy-efficient light sources with ultra-wideband properties ranging from visible to mid-IR, potentially revolutionizing the fields of information processing [7, 8], time–frequency metrology [3, 4], sensing [9] and quantum photonics [2].

**Figure 7:**
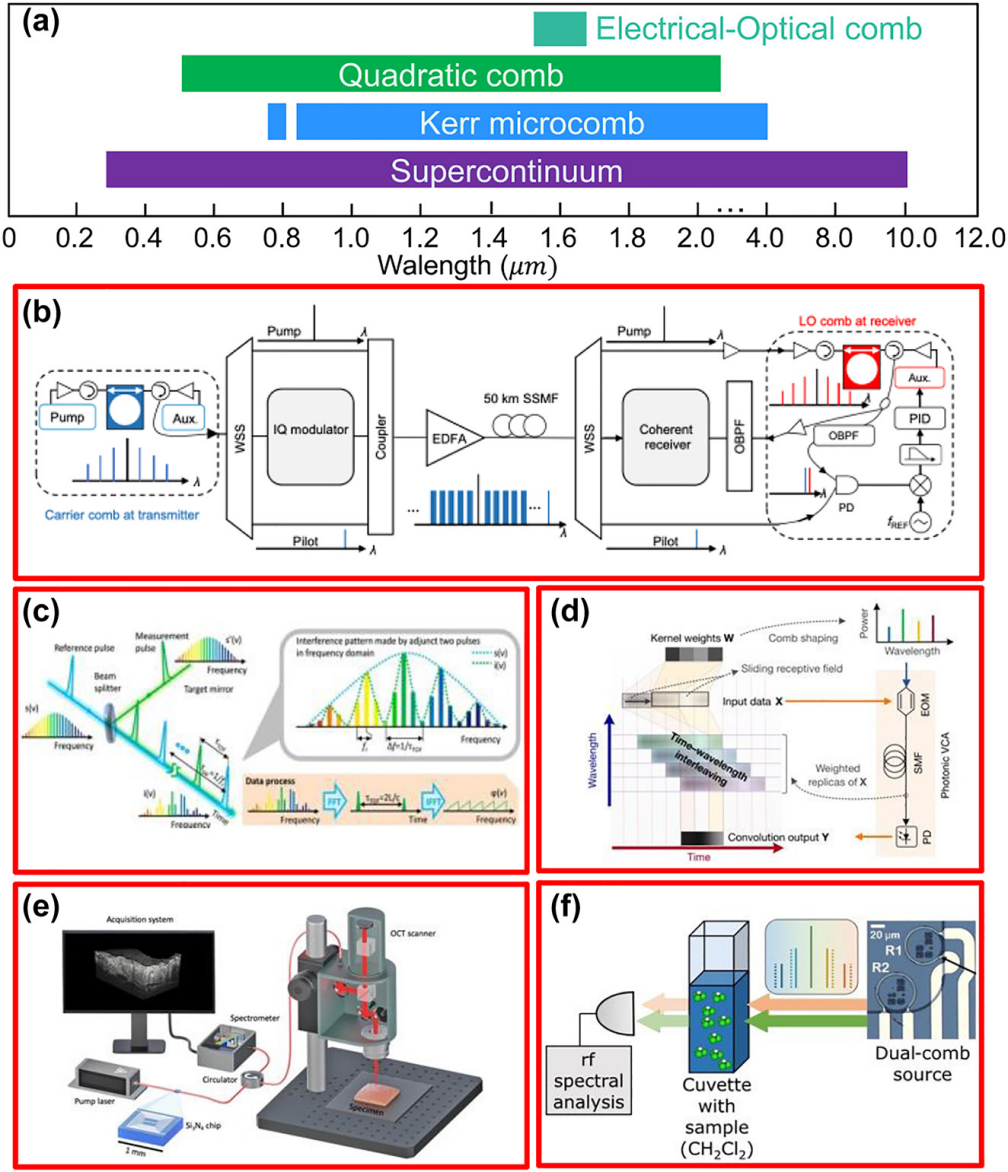
Typical OFC applications. (a) Map of the spectrum coverage for representative works using different comb-generation approaches (strips). (b) Optical interconnect using coherence-cloned DKS microcombs as carriers and Los [274]. (c) Architectural approach of the spectrally resolved ranging via soliton microcomb [247]. (d) Optical vector convolutional accelerator powered by a soliton crystal microcomb [7]. (e) Spectral domain OCT enabled by the generated SCG [225]. (f) Dual-comb spectroscopy using two microresonator combs generation [67]. [(b) Reproduced with permission from reference [274]. Copyright © 2022, nature publishing group. (c) Reproduced with permission from reference [247]. Copyright © 2021, American physical society. (d) Reproduced with permission from reference [7]. Copyright © 2021, nature publishing group. (e) Reproduced with permission from reference [225]. Copyright © 2021, AAAS. (f) Reproduced with permission from reference [67]. Copyright © 2018, AAAS].

OFCs are usually realized by optically pumping the optical media with strong nonlinearity, generating a phase-coherent light source with spectra of discrete, evenly spaced narrow optical laser lines [235]. The OFCs’ bandwidths can be extremely broad thanks to the nonlinear optical interactions. These interactions can generate a broad range of new optical frequencies significantly beyond those in the initial laser field incident on the nonlinear medium. Starting from the traditional mode-locked laser (MLL) [236], OFCs have evolved into one of the most active areas in photonics. The most remarkable examples of generating such broad spectra light sources are supercontinuum generation (SCG) in optical waveguides, Kerr-comb generation (KCG) through Kerr nonlinear process *χ*^(3)^ in microresonators [51, 165, 169, 237], [238], [239], [240], [241], [242], [243], [244], [245], [246], [247], [248], [249], [250], [251], [252], [253], [254], [255], [256], [257], [258], [259], [260], [261], [262], electro-Optical combs in modulators and quadratic combs through cascaded second harmonic generation *χ*^(2)^ process or *χ*^(2)^-based spectrum translation. Vast reviews in the OFCs can be found [263, 264]. Due to the abundant nonlinearity of wide bandgap semiconductors, both silicon-family related materials (from silica [265–267], Si_3_N_4_ [86, 245, 268], to silicon carbide (SiC) [269]), and non-group IV materials on silicon platform (i.e. 2D materials [165], Ta_2_O_5_ [91], AIN [270], LN,) have been utilized toward realizing chip-scale OFCs to generate hundreds of frequencies spanning tens of terahertz range from visible to mid-IR. Most of the aforementioned OFC demonstrations focus on the near-IR and mid-IR ranges, which are now relatively mature. However, visible OFCs are limited by strong material dispersion and high losses at short wavelengths. One way proposed to overcome these obstacles is to generate a parametric frequency comb in the near-IR and then convert it into visible region through second harmonic generation *χ*^(2)^ [270, 271]. Another approach is to use high-order mode to tailor the material dispersion [272]. Supercontinuum light generation has also shown promise for ultra-wideband frequency generation down to visible. The spectrum coverage for representative works using different comb-generation approaches is shown in Figure 7(a).

Benefiting from the excellent characteristics of OFCs including high repetition rate, high coherence, and wide frequency spectrum, silicon-based soliton microcomb devices have unique advantages in communications, ranging, microwave generation [254, 258, 273], spectroscopy, and quantum applications [2]. Here, we show some examples. For optical communication, Geng et al. cloned and regenerated the soliton micro comb 50 km away through pump laser transmission and two-point locking, and used them as the local oscillators (LOs) of the receiver to retain the original frequency and phase characteristics, as shown in Figure 7(b). In the acquisition of coherent data demodulation, the steps of electrical frequency offset estimation and carrier phase estimation based on digital signal processing can be greatly simplified, and terabit coherent data interconnection can be realized [274]. For optical ranging and metrology, Jang et al. reported spectrally resolved laser dimensional metrology via a free-running soliton frequency microcomb with nanometric-scale precision, by employing a hybrid timing signal from comb-line homodyne, microcomb, and background amplified spontaneous emission spectrally resolved interferometry [247], as shown in Figure 7(c). Li et al. unraveled the transitional dynamics of frequency microcombs from chaotic background routes to femtosecond mode-locking in real time [259]. For optical computing, Moss et al. demonstrated that the exciting photonic neural network processor using microcomb parallelization holds promise in surpassing the speed and energy efficiency of cutting-edge graphics processing units [7]. Powered by a soliton crystal microcomb (spacing of ∼48.9 GHz) with 90 wavelengths occupying 36 nm across the C-band, the universal optical vector convolutional accelerator (see Figure 7(d)) operating at more than 11 Tera Operations Per Second (TOPS)has been achieved, demonstrating convolutions of images with 250,000 pixels for facial image recognition and 88 per cent accuracy for handwritten digit images recognition. For optical spectroscopy, Ji et al. [225] demonstrated a SCG platform based on a 1-mm^2^ Si_3_N_4_ photonic chip for spectral domain OCT, as shown in Figure 7(e). The generated SCG has a flat 3-dB bandwidth of 105 nm near 1.3 µm without any postfiltering. The image breast tissue has been used to demonstrate strong imaging performance with 105-dB sensitivity and 1.81-mm 6-dB sensitivity roll-off with 300-μW optical power on sample. Dual-comb spectroscopy (DCS) uses heterodyne beating between two frequency combs with slightly different repetition rates to generate a sequence of beat notes, which down-converts the spectral information spanning tens to hundreds of terahertz in the optical domain to a few GHz in the radio frequency (RF) domain. DCS is a powerful technique for real-time, broadband optical sampling of molecular spectra with a high signal-to-noise ratio (SNR) and tens-of-microsecond acquisition times, which requires no moving components. Avik et al. [67] demonstrated the simultaneous generation of two microresonator combs for DCS with broadband optical spectra spanning 51 THz on the same chip from a single laser, and achieved high SNR absorption spectroscopy spanning 170 nm over a 20-ms acquisition time, as shown in Figure 7(f).

Along the way, we expect that integrated OFCs will find new markets in various important applications from visible to mid-IR. In the near-IR region, silicon integrated frequency comb devices bridging the optical band and the microwave band have shown progressing mesoscopic and macroscopic applications beyond aforementioned, such as high precision spectroscopy [3, 4], sensing [9], computing [7, 8] and quantum entanglement [2]. The recently developed CMOS-foundry-based visible photonic platform leveraging wideband gap semiconductors for next generation silicon photonics [203] could possibly extend OFCs to novel wavelength ranges, such as the visible region thus bringing new opportunities in areas like atomic physics and biosensing.

### 3.3 Integrated quantum technologies

Quantum photonic technologies have found broad application scenarios, from communication [275] and secure key distribution [276], computing [277], to quantum metrology [278], etc. Recent advances in integrated quantum photonics [1, 279] show great promise for chip-scale quantum information processing with tremendous efficiency yet low complexity and are being intensively investigated. However, low-loss storage, transmission and computing of quantum information systems, such as trapped ions, neutral atoms, and spins in crystals are connected to photons in the UV, visible, or short near-IR bands [280]. Ultra-wideband integrated photonic devices on silicon platforms are promising candidates to enable quantum technologies.

**Figure 8:**
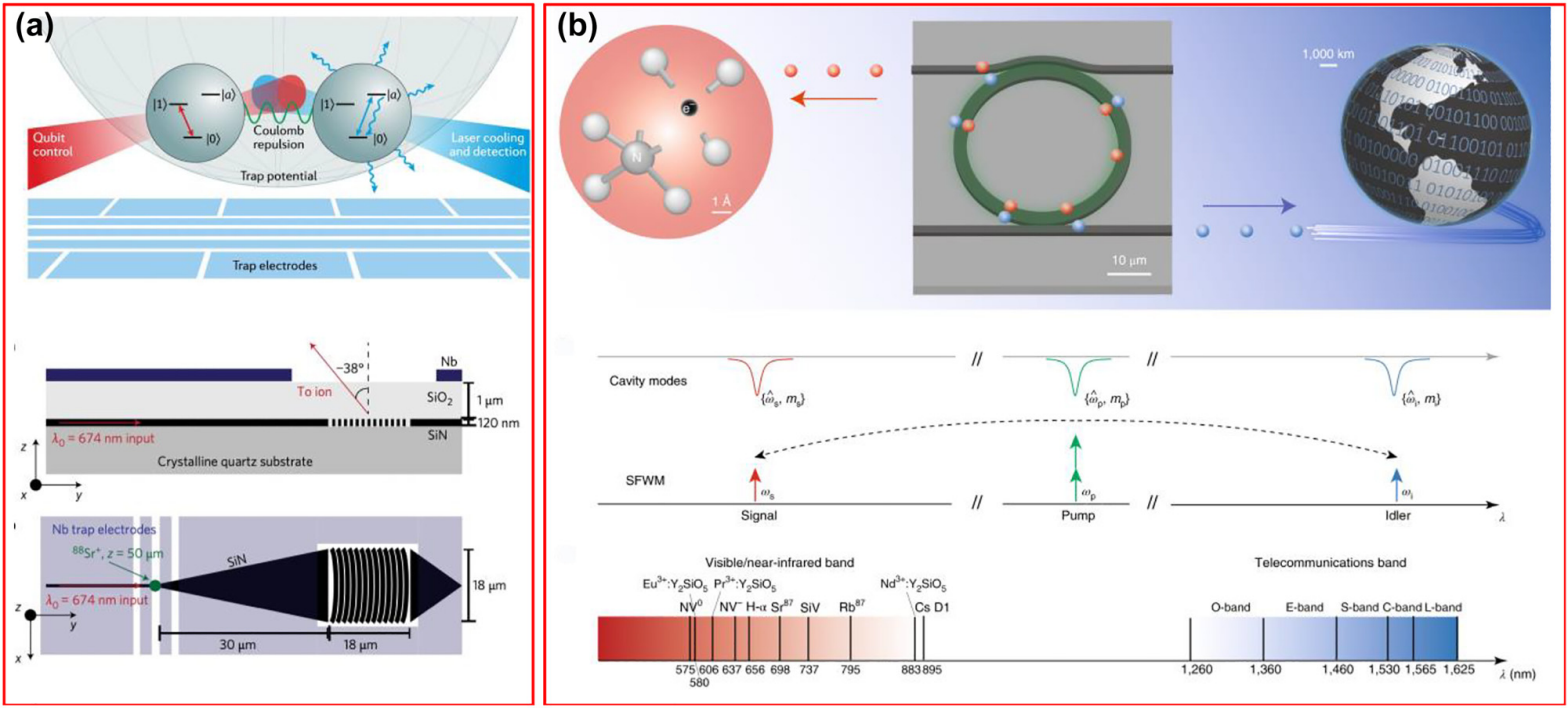
Integrated quantum technologies. (a) Quantum computing with trapped ions [281]; focusing grating schematic to deliver 674 nm light to Sr^+^ ions [282]. (b) Chip-integrated visible–telecom entangled photon pair source for quantum communication [284]. [(a) Reproduced with permission from reference [281]. Copyright © 2021, nature publishing group; reproduced with permission from reference [282]. Copyright © 2016, nature publishing group. (b) Reproduced with permission from reference [284]. Copyright © 2019, nature publishing group].

Trapped-ion quantum (QIP) information processing [281] is among the most promising systems for quantum computing, which stores and manipulates information in atomic ions’ internal and shared motional quantum states maintained in position in free space by electric fields using optical and microwave signals. Figure 8(a) shows a schematic overview of QIP. The qubits in a trapped ion quantum computer are the internal electronic states of individual atomic ions. The ions are held in an electromagnetic trap. Lasers or microwaves are used to control the qubit states |0⟩ and |1⟩. The internal control and the Coulomb repulsion between ions combine to form conditional logic gates. Readout is performed by measuring laser induced ion fluorescence using an auxiliary state |*a*⟩. The laser-induced fluorescence is also used to cool the ions and prepare for quantum logic. The challenges of optical materials arise in the generation of electromagnetic traps, in the control of laser beams and in the detection of ions’ fluorescence. For the typical atomic structure of the ion species used in QIP, the key requirement for optical materials is high transmittance over a wide wavelength range (0.3–2 μm) for full trapped-ion control and readout, especially the near-UV wavelengths required by many commonly used ions. In addition, the integrated photonic devices with low loss must be developed using materials that can be fabricated with low roughness, because the loss induced by Rayleigh scattering scales poorly with the decrease of wavelength. Recently, Si_3_N_4_ waveguides, grating couplers, with silicon dioxide cladding have been integrated into a surface-electrode trap and successfully used to deliver 674 nm light to Sr^+^ ions [282], which shed blue light to other wide bandgap materials transparent from UV-to-visible wavelength range on silicon, such as Si_3_N_4_ [203], GaN [141], AlN [212], LN [109] and Al_2_O_3_ [90]. In addition to light delivery, the beams generally need to be switched on and off using integrated optical modulators with large EO or piezoelectric (PE) coefficients in the UV-to-visible wavelength range [279], which again can leverage the wide bandgap photonics that are transmissive over the wavelength regions of interest for trapped ions on silicon platform. Therefore, GaN [141], AlN [212] and LN [109] are the three most promising materials.

Photon pairs are the fundamental building blocks for quantum entanglement and quantum communication. Silicon photonics has demonstrated the generation of photon pairs within the telecommunications band [283]. However, the high optical attenuation of silica in the visible or short near-IR band limits the length scale of the fiber-based quantum communication between local node operations. One promising way to generate visible–telecom photon pairs is using a wide bandgap material for high Q resonators through spontaneous four-wave mixing (SFWM), as shown in Figure 8(b). Srinivasan et al. [284] have demonstrated visible–telecom time–energy entanglement in the high Q Si_3_N_4_ resonator with distribution over a 20-km fiber, which is far exceeding the efficient fiber length that purely visible wavelength quantum light sources can propagate. Further, using other wide bandgap materials covering visible to short near IR, such as GaN [141], AlN [212], LN [109] and Al_2_O_3_ [90] on silicon platform, together with the dispersion engineering of the microresonators, may enable conversion of quantum information between different species of trapped atoms/ions, defect centers, and quantum dots to the telecommunications bands for future quantum communication systems.

## 4 Conclusion and perspective

Although silicon photonics on SOI platform has achieved tremendous success in the telecom band, appealing platforms that support wafer-scale and heterogeneous integration of wide bandgap materials, like Si_3_N_4_, LN, Al_2_O_3_, AlN, and Ta_2_O_5_, are highly desired for emerging applications such as biochemical sensing and OCT [285], precise metrology and spectroscopy [3, 4], quantum optics [1, 2], RGB displays [5], atomic clocks [6], computing [7, 8], remote sensing [9], etc. These wide bandgap materials possess key performance features that are not available in SOI, including the wide transparency window from visible to mid-IR, ultralow linear, and nonlinear losses and high-power handling capability.

Here, we have reviewed some representative studies and the latest progress of these wide bandgap materials on the silicon platform. The demonstrated performance improvements are remarkable, reaching or exceeding the performance of current SOI platforms in passive, active or nonlinear applications over a wide wavelength range from visible to mid-IR. However, fully utilizing these wide bandgap materials on the silicon platform is still in the early stage of development, and we expect to see more developments in the future. Further work needs to reduce the waveguide loss in visible and mid-IR as they are still relatively high compared with those works reported in near-IR. Firstly, from an economic point of view, the wafer size of the wide bandgap materials on the silicon platform should be gradually increased to 8, 10, and 12 inches to allow for a large-scale fabrication. At present, only Si_3_N_4_ and LNOI platforms have been proved to be capable of producing large wafers. Secondly, it is still difficult to integrate electrically pumped light sources with these wide bandgap materials on silicon. III–V compound semiconductor devices are highly desired for on-chip laser sources. Substantial research has been devoted to solve this problem through hybrid/heterogeneous integration techniques [286, 287]. Thirdly, highly efficient on-chip detectors in the mid-IR range are still in the early stage. Germanium-on-silicon (or SiGe-on-Si) is a very exciting wideband material platform for detection with CMOS processing compatibility [288]. Because the direct energy gap is 0.8 eV, Ge can effectively absorb optical radiation above 1.6 µm. High-quality Ge can be epitaxially grown on silicon, and the high refractive index of Ge means that the silicon substrate can be used as the bottom cladding, which allows the transparency window to be extended to approximately 7–8 μm. In addition, Ge has a higher nonlinearity than silicon, which makes it ideal for nonlinear applications. Currently, the material quality is a challenge that hinders Ge films from achieving low losses. Finally, the integration PIC platform to fully support visible to mid-IR applications is still absent. The 3D (vertical) integration of different wide bandgap materials may be a promising route.

**Figure 9:**
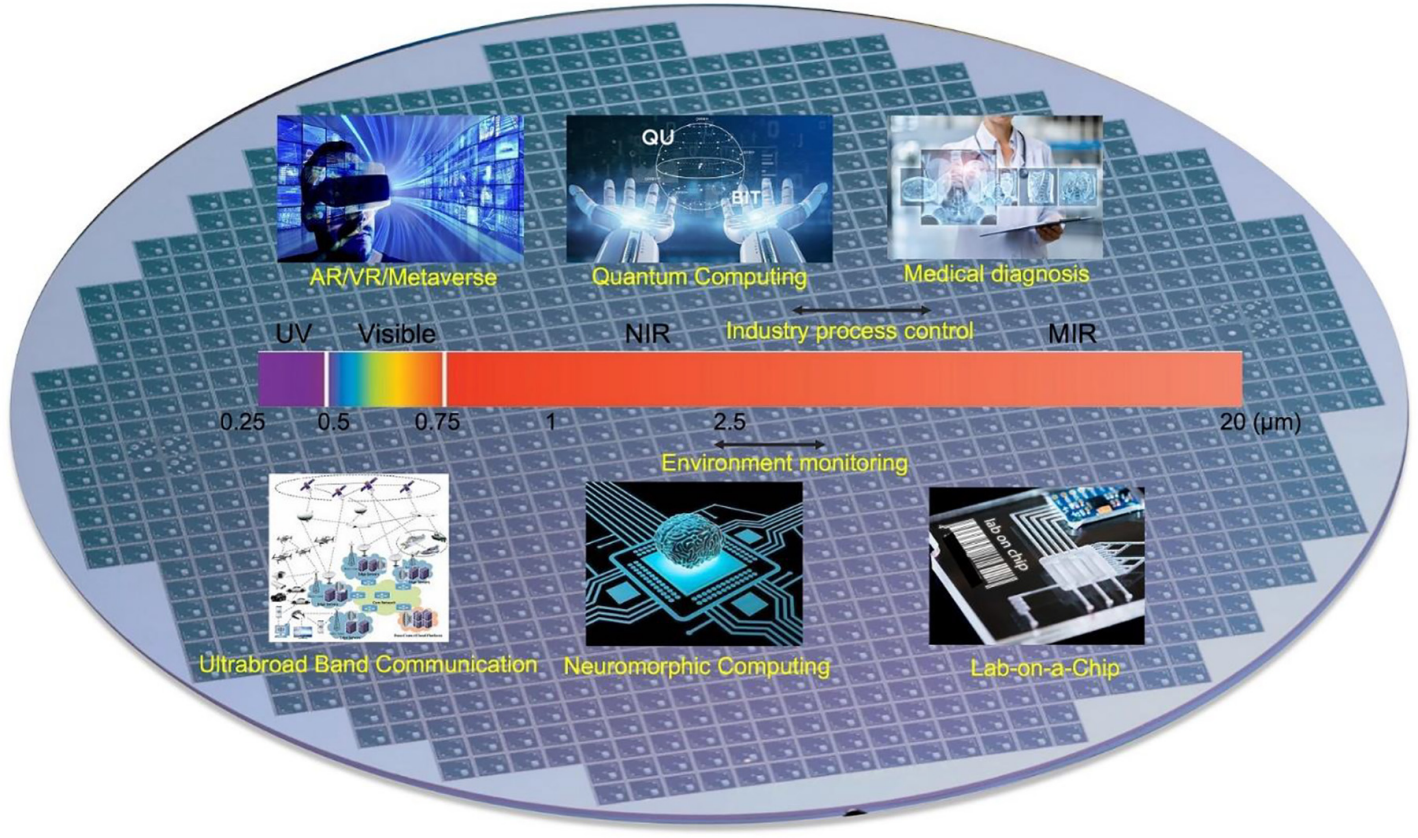
Future application outlook of wideband spectrum from visible to mid-IR.

We believe that future technologies will cover the wide wavelength range from visible to mid-IR to address the diverse applications. Various high-performance passive, active, linear, and nonlinear components can be integrated on-chip. As shown in Figure 9, from UV to MIR, we expect to see rapid progress in the fields of ultra-broadband communication, sensing, AR/VR/Metaverse, quantum computing, neuromorphic computing, medical diagnosis, lab-on-a-chip, and so on.
